# Genome-Wide Investigation of the Zinc Finger-Homeodomain Family Genes Reveals Potential Roles in Apple Fruit Ripening

**DOI:** 10.3389/fgene.2021.783482

**Published:** 2022-01-17

**Authors:** Xian-bo Zheng, Yao Wu, Hao Wang, Shang-wei Song, Tuan-hui Bai, Jian Jiao, Chun-hui Song, Hong-guang Pang, Miao-miao Wang

**Affiliations:** College of Horticulture, Henan Agricultural University, Zhengzhou, China

**Keywords:** apple, ZF-HD, genome-wide, gene expression, fruit ripening

## Abstract

Zinc finger-homeodomain (ZF-HD) transcription factors play an important role in the regulation of plant growth and development, as well as the regulation of stress responses. Studies on the ZF-HD family genes have been conducted in many plants, however, the characteristics of this family in apple (*Malus domestica*) fruit remains to be poorly understood. In this study, we identified nineteen *ZF-HD* family genes in apple at the whole-genome scale, which were unevenly located on ten chromosomes. These *MdZF-HD* genes were phylogenetically divided into two subfamilies: zinc finger-homeodomain (ZHD) and MINI ZINC FINGER (MIF), and the ZHD subfamily was further classified into five groups (ZHDI–ZHDV). Analysis of the gene structures showed that most *MdZF-HD* genes lack introns. Gene expression analysis indicated that nine selected *MdZF-HD* genes were differentially responsive to 1-MCP (1-methylcyclopropene) treatment during the postharvest storage of “Qinguan” apple fruit. Moreover, the transcripts of six genes were further validated in “Golden Delicious” apple fruit, and five genes (*MdZHD1/2/6/10/11*) were significantly repressed and one gene (*MdZHD7*) was slightly induced by ethylene treatment. These results indicated that these six *MdZF-HD* genes may involve in the regulation of ethylene induced ripening process of postharvest apple fruit. These findings provide new clues for further functional investigation of *ZF-HD* genes, such as their roles in the regulation of fruit ripening.

## Introduction

Varieties of regulatory proteins manipulate a series of developmental processes in plants. Among them, transcription factors (TFs) have been shown to play important roles in regulating the different biological processes of plant growth, flowering, fruiting, and fruit ripening (Mitsuda and Ohme-Takagi, 2009). Recently, zinc finger-homeodomain (ZF-HD), a plant specific transcription factor family, has attracted increasing attention due to its role in regulating plant growth and development as well as in response to a variety of biotic and abiotic stresses (Agarwal and Jha, 2010; Zhang et al., 2015; Khatun et al., 2017). ZF-HD transcription factors are mainly composed of a N-terminal C2H2-type zinc finger domain (ZF) and a C-terminal homeodomain (HD) domain (Tran et al., 2006). According to phylogeny, the ZF-HD gene family can be divided into zinc finger-homeodomain (*ZHD*) and MINI ZINC FINGER (*MIF*) subfamilies (Vision et al., 2000; Schoof et al., 2004). The MIF proteins contain the ZF domain but lack the HD domain (Hu and Ma, 2006), and the evolutionary relationship of ZHDs and MIFs remain unclear. Zinc finger protein has a local polypeptide structure and is formed by cysteine/histidine binding zinc ions (Tan and Irish, 2006). The ZF domain is rarely involved in DNA binding, but can enhance the protein-DNA interactions mediated by the HD domain (Windhövel et al., 2001). HD is a DNA binding domain, which is composed of a highly conserved basic sequence with a length of about 180 bp and can encode 60 amino acid sequences (Mukherjee et al., 2009). HD proteins are mostly correlated to other domains or motifs as well as protein-protein interactions (Ariel et al., 2007).

Currently, ZF-HD has been reported in many plant species, after the first discovery in the C4 plant *Flaveria trinervia* (Windhövel et al., 2001). For example, seventeen *ZF-HD* genes were identified in the model plant *Arabidopsis thaliana* (Hu et al., 2008), 31 members in Chinese cabbage (Wang et al., 2016), 22 members in tomato (Khatun et al., 2017), 37 members from cotton (Abdullah et al., 2018), 20 members from bitter buckwheat (Liu et al., 2019), 18 members in tea tree (Zhou et al., 2021), and 10 members from cucumber (Lai et al., 2021). Several studies show that *ZF-HD* genes can act as regulators in response to various stresses as well as during flower or fruit development (Tan and Irish, 2006; Zhou et al., 2021). In Arabidopsis, *ZFHD1* can be induced by drought, high salinity, low temperature and abscisic acid (ABA), and can also bind to the promoter of *ERD1* (*EARLY RESPONSE TO DEHYDRATION STREES 1*) gene (Tran et al., 2006). In soybean, *GmZF-HD1* and *GmZF-HD2* were up-regulated in response to pathogen infection and can bind to the promoter of *GmCaM4* gene (Park et al., 2007). In rice, the overexpression of *OsZF-HD1* gene leads to curl and drooping of rice leaves (Xu et al., 2014). In *Camellia sinensis*, the transcription level of *CsZF-HD5* is very high in flower tissues, suggesting that *CsZF-HD5* is closely related to flower development (Zhou et al., 2021). Furthermore, four *SlZHD* genes (*SlZHD1/19*/*20*/*22*) were highly expressed in mature tomato fruit (Khatun et al., 2017). Although *ZF-HD* family genes have been widely investigated in several model plants and some other species, the comprehensive analysis of *ZF-HD* family in apple has rarely been reported.

Apple (*Malus domestica*) is an important economic crop cultivated worldwide. As a climacteric fruit, the ripening process of apple fruit is highly dependent on ethylene (Yue et al., 2020). The rates of postharvest ripening and softening process critically impact the shelf life of apple fruit. Therefore, it is of significance to study the molecular mechanisms of the ripening process for apple fruit. In this paper, a genome-wide analysis of the *MdZF-HD* genes based on the apple genome data was conducted to explore their potential roles in regulating the postharvest ripening process. The phylogenetic relationships, gene structures, chromosome locations and replication events of the *ZF-HD* genes in apple were introduced in detail. Furthermore, the expression profiles of *MdZF-HD* genes in response to ethylene or 1-MCP (1-methylcyclopropene, the ethylene receptor inhibitor) treatment in apple fruit were analyzed by quantitative real time PCR (qRT-PCR) technique. This study is expected to provide valuable clues for the functional investigation of *ZF-HD* family genes in the regulation of apple fruit ripening.

## Materials and Methods

### Plant Materials and Treatments

Two cultivars of apple (*Malus domectica*) fruit were selected, including one late-ripening cultivar “Qinguan” and one mid-ripening cultivar “Golden Delicious.” Mature “Qinguan” apple fruit were harvested from a commercial orchard at Lingbao (Henan, China) in 2018. Each picked fruit was inspected to be free from mechanical damages, diseases and insect pests. The fruits were divided into two batches for two different treatments. Each batch contained three replicates of approximately 270 fruits. The fruits were treated with 1-MCP (1 μL L^−1^, 20°C, 24 h), or air as the control group (20°C, 24 h) in 25-L airtight containers. The weight of apple fruit in each containers was about 6 kg. For the 1-MCP fumigation treatment, 1.22 mg 1-MCP powder (effective mass fraction is 3.30%) was dissolved in 1 mL distilled water about 40°C in 1.5 mL centrifuge tube. The tube containing the 1-MCP reagent was put to the bottom of the containers, and the lid was opened exactly before the containers was sealed.

To verify the effect of 1-MCP treatment, and to confirm the roles of ethylene in fruit ripening, mature “Golden Delicious” apple fruit were collected from a commercial orchard at Luoning (Henan, China) in 2019. The 1-MCP treatment were the same as in 2018, and the fruits were treated with ethylene (100 μL L^−1^, 20°C, 24 h), with those treated in air as control group (20°C, 24 h) in 25-L airtight containers. The fruits after treatment were transferred to storage in air with relative humidity of 85–90% at 20°C. The sampling points were 0, 7, 14, 21 and 28 days, respectively.

At each sampling time, twelve fruits from three replicate samples (four fruits in each) were collected from each batch. The outer pericarp (without skin) were cut into pieces and immediately frozen in liquid nitrogen and then stored at −80°C until future use.

### Identification of *ZF-HD* Genes in Apple Fruit

Apple genome annotation information and genome sequence were sourced from the Rosaceae genome website GDR (https://www.rosaceae.org/). The HMM (Hidden Markov Model) configuration profiles of ZF-HD (PF04770) was downloaded from the Pfam 34.0 database (https://pfam.xfam.org/) and perform sexual screening through *e* value < 0.01. The molecular mass, isoelectric point and other physical and chemical properties of the identified nineteen apple ZF-HD proteins were obtained by using the tools of the ExPASy 3.0 (https://web.expasy.org/compute_pi/) website (Duvaud et al., 2021). The prediction of subcellular location on the Cell-PLoc 2.0 (http://www.csbio.sjtu.edu.cn/) was also conducted (Chou and Shen, 2008).

### Phylogenetic Tree Analysis of the Apple *ZF-HD* Gene Family

The protein sequences of the Arabidopsis ZF-HD family were downloaded from the PlantTFDB database v5.0 (http://planttfdb.gao-lab.org/) and the MEGAX (v. 10.2.4) software was used to construct a phylogenetic tree by the Neighbor-joining (NJ) method {Formatting Citation} (Jin et al., 2017; Kumar et al., 2018). The evolution standard Bootstrap value is 1000. The evolutionary tree is optimized by EvolView v2 (https://www.evolgenius.info/evolview/) (He et al., 2016).

### Analysis of Apple ZF-HD Conserved Motifs and Gene Structures

The conserved motifs of the apple ZF-HD proteins were analyzed by Multiple Em for Motif Elicitation (MEME) online server (version 5.3.3, https://meme-suite.org/meme/tools/meme) (Bailey et al., 2009). The structures of the apple *ZF-HD* genes were visualized using TBtools software (Chen et al., 2020). Besides, the arrangement of the introns and exons of the nineteen *MdZF-HD* genes were obtained visually.

### Chromosomal Locations and Collinearity Analysis of the Apple *ZF-HD* Genes

The apple chromosome file information and the GFF file configuration information were used to obtain the chromosome interval information of the apple ZF-HD gene family. The visualization was achieved by TBtools. The Genome Collinearity Analysis Toolkit (MCScanX) was used to analyze the collinearity between each pair of chromosomes (Wang et al., 2013).

### Analysis of *Cis*-elements of the Apple *ZF-HD* Promoter

The 2000 bp upstream sequence of the start codon (ATG) of each *MdZF-HD* gene was extracted from the *Malus domestica* genome database (GDDH13 1.1, https://iris.angers.inra.fr/gddh13/the-apple-genome-downloads.html) (Daccord et al., 2017) and submitted to the PlantCARE server (http://bioinformatics.psb.ugent.be/webtools/plantcare/html/) to analyze the distribution of *cis*-elements of the ZF-HD family genes (Lescot et al., 2002).

### RNA Extraction, cDNA Synthesis, and Real-Time PCR Analysis

Total RNA was extracted from frozen fruit flesh (1.0 g) by the method described by Wang et al. (2021). The PrimeScript^TM^ RT reagent Kit with gDNA Eraser (TaKaRa) was used to remove the contaminated gDNA. cDNA was synthesized from 1.0 µg DNA-free RNA, using the Reverse Transcription System (TaKaRa). At each sampling point, three biological replicates were performed for RNA extraction.

Oligonucleotide primers used for real-time quantitative PCR analysis were designed with Primer3 (version 0.4.0, https://bioinfo.ut.ee/primer3-0.4.0/). Gene specificity of the primers was determined by melting curves and PCR products resequencing. The sequences of primers used for PCR analysis are listed in Supplementary Table S1. The apple *Actin* gene, a housekeeping gene, was chosen to monitor the abundance of mRNA (Wang et al., 2021).

Real-time PCR was performed using an ABI PRISM 7500 instrument (Applied Biosystems). The PCR protocols were the same as our previous reports (Wang et al., 2021), with SYBR^TM^ Select PCR Master Mix (Applied Biosystems). The relative expression levels of genes were calculated by the 2^- ∆∆Ct^ method (Livak and Schmittgen, 2001).

### Subcellular Localization Analysis

The full-length coding sequences of three selected *ZF-HD* genes (*MdZHD2/6/7*) without the stop codons were amplified with primers (described in Supplementary Table S2) and constructed into the GFP vector (Li S. J. et al., 2017). 35S-*MdZHD2*-GFP, 35S-*MdZHD6*-GFP and 35S-*MdZHD7*-GFP were transiently expressed in tobacco (*Nicotiana benthamiana*) leaves by *Agrobacterium*-mediated infiltration (GV3101) (Zeng et al., 2019). The tobacco plants were grown in an artificial climate room at 22°C with daylight extension to 16 h. The green fluorescent protein (GFP) fluorescence of tobacco leaves was imaged 3 days after infiltration using the Laser Scanning Confocal Microscope (Nikon, AIR HD25, Japan).

### Statistical Analysis

Statistical significance of differences were calculated using the Student´s *t*-test by DPS 7.05 (Zhejiang University, Hangzhou, China). Figures were drawn with P_RISM_ 8 (Graphpad, San Diego, CA, United States).

## Results

### Identification and Classification of *ZF-HD* Family Genes in Apple

Nineteen *ZF-HD* family candidate genes were finally obtained by using the NCBI conserved domain database *CDD* and *smart* website for double verification of the conserved structure of the protein, and the genes were named *MdZHD1*-*MdZHD15* and *MdMIF1*-*MdMIF4* based on the chromosome location information (Table 1). The CDS lengths of the family members ranged from 273 bp (*MdMIF2/4*) to 1131 bp (*MdZHD13*). The lengths of MdZF-HD proteins were 90–376 amino acids (AA), and the molecular weight (MW) varied from 9.62 to 41.65 KDa. Besides, the predicted isoelectric points (pIs) of MdZF-HD proteins ranged from 6.51 (MdZHD12) to 9.44 (MdZHD2). The results indicated that except for the acidic proteins (MdZHD5/11/12/15), the other MdZF-HD proteins were basic proteins. The subcellular localization prediction showed that all the MdZHD proteins (MdZHD1-15) were located in the nucleus, while the MdMIF proteins (MdMIF1-4) were located in the mitochondria. This is consistent with the previous research that most ZF-HD proteins located in the nucleus (Wang et al., 2016).

**TABLE 1:** Characteristics of *ZF-HD* gene identified in apple.

Sequence ID	Gene name	Chr	CDS (bp)	AA	MW (kDa)	pI	Genomic location	Subcellular localization
MD01G1037200	MdZHD1	1	849	282	30.45	8.57	12886256–12888351	Nucleus
MD02G1146300	MdZHD2	2	963	320	35.27	9.44	12084535–12085497	Nucleus
MD06G1234500	MdZHD3	6	609	202	22.04	7.67	36543187–36543795	Nucleus
MD08G1030700	MdZHD4	8	1,038	345	37.32	7.09	2193400–2195131	Nucleus
MD08G1192300	MdZHD5	8	798	265	28.70	6.57	24640151–24642134	Nucleus
MD09G1051200	MdZHD6	9	1,062	353	38.44	7.12	3408795–3409856	Nucleus
MD13G1072300	MdZHD7	13	1,002	333	36.30	9.30	5088637–5089638	Nucleus
MD14G1241400	MdZHD8	14	606	201	21.95	8.48	32002796–32003401	Nucleus
MD15G1025900	MdZHD9	15	1,026	341	36.46	7.33	1509804–1511909	Nucleus
MD15G1260600	MdZHD10	15	936	311	34.12	9.19	22228452–22229387	Nucleus
MD15G1316100	MdZHD11	15	837	278	29.53	6.99	32327019–32327855	Nucleus
MD15G1380900	MdZHD12	15	831	276	29.86	6.51	46856040–46859412	Nucleus
MD15G1443500	MdZHD13	15	1,131	376	41.65	7.05	54366137–54367267	Nucleus
MD16G1072800	MdZHD14	16	969	322	35.07	9.17	5118380–5119348	Nucleus
MD17G1051300	MdZHD15	17	1,047	348	37.90	6.59	3826736–3827782	Nucleus
MD06G119320	MdMIF1	6	297	98	11.00	8.92	232853856–3285415	mitochondrial
MD09G1051100	MdMIF2	9	273	90	9.618	8.23	3395078–3395350	mitochondrial
MD14G1200200	MdMIF3	14	285	94	10.50	8.77	28972850–28973134	mitochondrial
MD17G1051200	MdMIF4	17	273	90	9.662	7.63	3796444–3796716	mitochondrial

### Phylogenetic Classification, Genetic Structure Analysis, and Conserved Motif Analysis of the *ZF-HD* Family Genes in Apple

**FIGURE 1:**
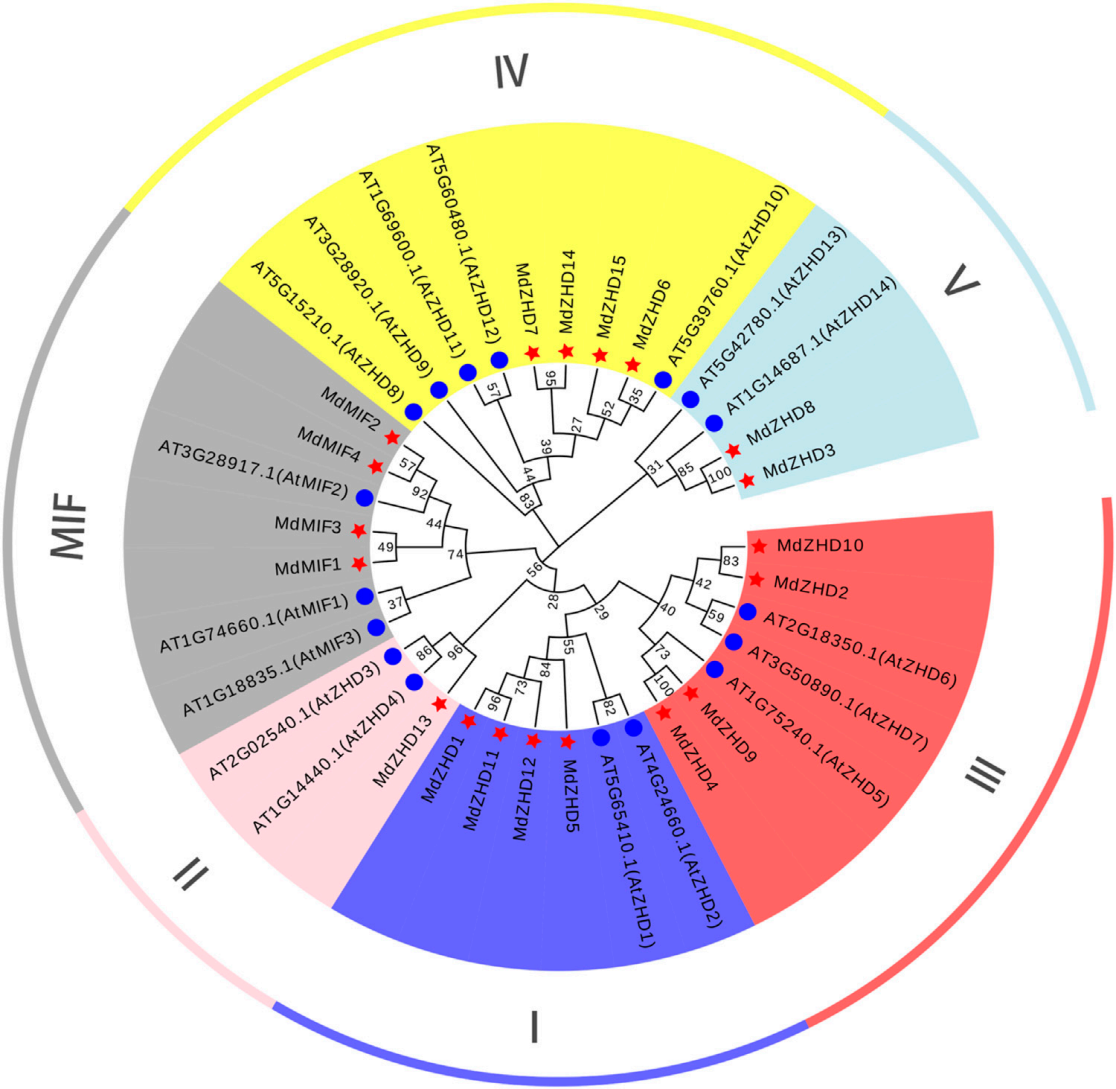
The phylogenetic tree of ZF-HD proteins from apple (*Malus domestica*, MdZHD) and Arabidopsis (*Arabidopsis thaliana*, AtZHD). The tree was constructed by the Neighbor-joining (NJ) method using a MEGAX software with 1000 bootstrap replications. The MdZHD member was accompanyed with a red pentagram. Six subfamilies were identified in apple and were distinguished in different color: ZHD I, ZHD II, ZHD III, ZHD IV, ZHD V, and mini zinc finger (MIF).

To gain insights into the evolutionary relationship of the ZF-HD family proteins in apple, a NJ phylogenetic tree consisting of Arabidopsis (17 genes) and apple (19 genes) was constructed (Figure 1). The sequences of protein used for phylogenetic tree analysis were listed in Supplementary Table S3. According to the ZF-HD family classification of Arabidopsis, the apple ZF-HD gene family was phylogenetically divided into two subfamilies: ZHD and MIF. ZHD was further divided into five parts, including ZHDI (*MdZHD1/5/11/12*), ZHDII (*MdZHD13*), ZHDIII (*MdZHD2/4/9/10*), ZHDIV (*MdZHD6/7/14/15*) and ZHDV (*MdZHD3/8*) (Figure 1). Among these, the ZHDII subfamily of apple has the least gene with only one.

**FIGURE 2:**
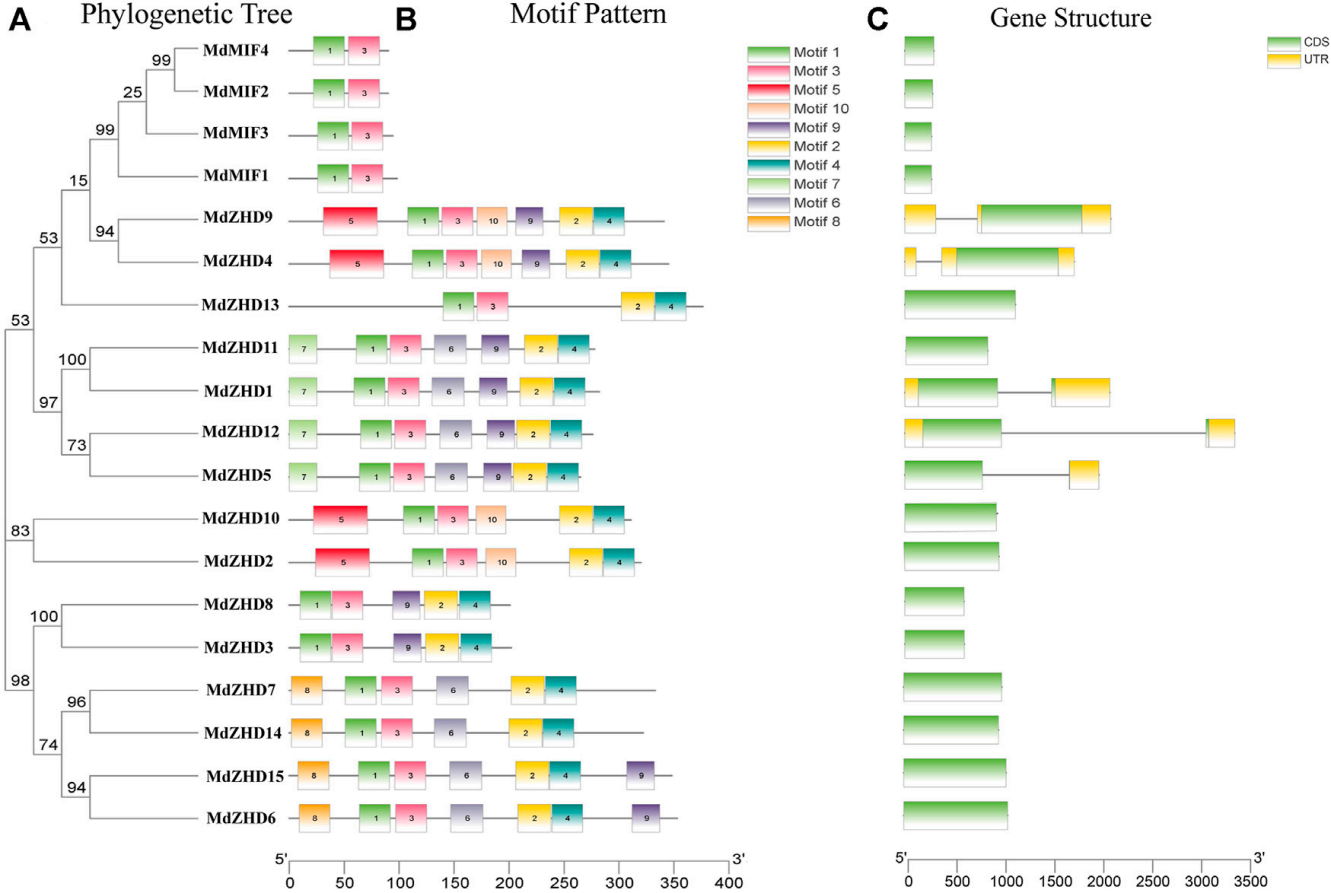
Phylogenetic relationships **(A)**, conserved motifs **(B)** and gene structures **(C)** of *MdZF-HD* genes. Different colored boxes represent different kinds of motifs. CDS sequences are represented by green rounded rectangles, introns are represented by gray lines, and UTRs are represented by yellow boxes.

In order to further investigate the diversity of the apple *ZF-HD* family genes, MEME web server was used to analyze the conserved motifs of the MdZF-HD proteins. From the results of the MEME analysis, ten conserved motifs were identified (Figure 2) (Supplementary Figure S1). All MdZF-HD proteins contain motif1 and motif3, indicating that motif1and motif3 are the specific motifs of the ZF-HD gene family (Figure 2B). It is worth noting that all members of the MdZHD subfamily contain motif1, motif2, motif3 and motif4, while the MdMIF proteins only contain motif1 and motif3. This result is consistent with the previous report (Hu and Ma, 2006; Hu et al., 2018) that the MIF subfamily harbors only a ZF domain but lacks HD (Supplementary Figure S2). Thus, motif2 and motif4 are the specific motifs for MdZHD subfamily and these motifs all appear in pairs. Besides, the types and numbers of motifs were identical between MdZHD4 and MdZHD9, MdZHD1 and MdZHD11, MdZHD2 and MdZHD10, MdZHD6 and MdZHD15, respectively. These results indicated that the ZF-HD members in the same subgroup contained the similar motifs. Furthermore, compared with the MdMIF subfamily, the different motifs existed among different members of the MdZHD subfamily supply evidence of their functional diversity. For example, MdZHD13 only had the motif1, motif2, motif3 and motif4, indicating that the MdZF-HD family genes differed in the evolutionary degree.

To further understand the composition of the *MdZF-HD* gene structure, we obtained the exon and intron structure of the gene through an annotation file. Among the nineteen *MdZF-HD* family genes, only five *MdZF-HD* genes contain introns, and each of them contains one intron (Figure 2C). It means that the function of the genes that lack introns is relatively conserved, which is consistent with the previous reports (Windhövel et al., 2001; Wang et al., 2016; Liu et al., 2019) that most *MdZF-HD* family genes lack introns.

### Chromosomal Localization and Collinearity Analysis of the *MdZF-HD* Family Genes

**FIGURE 3:**
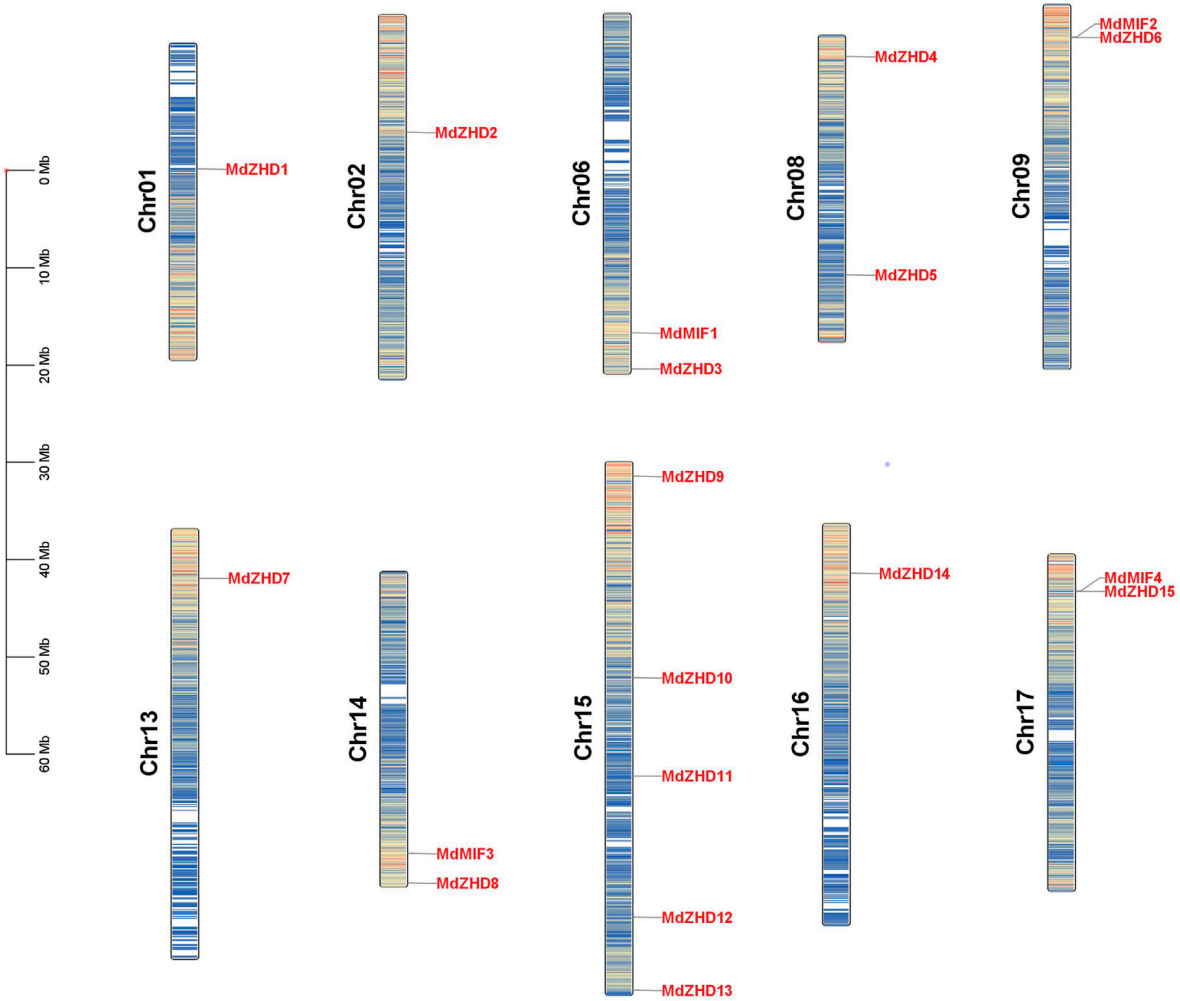
Distribution of *MdZF-HD* genes along apple chromosomes. The scale on the left stands for the length of each chromosome shown in mega base (Mb).

As shown in Figure 3, nineteen *MdZF-HD* genes were distributed on ten chromosomes in the apple genome. Chromosome 1 (Chr01), Chr02, Chr13 and Chr16 each had one *MdZF-HD* gene; Chr06, Chr08, Chr09, Chr14 and Chr17 contained two *MdZF-HD* genes, respectively; while Chr15 harbored the largest number of *MdZF-HD* genes (five genes) (Figure 3). The distribution of the genes was uneven. This suggests that the genes play an important role in different transcription initiations.

**FIGURE 4:**
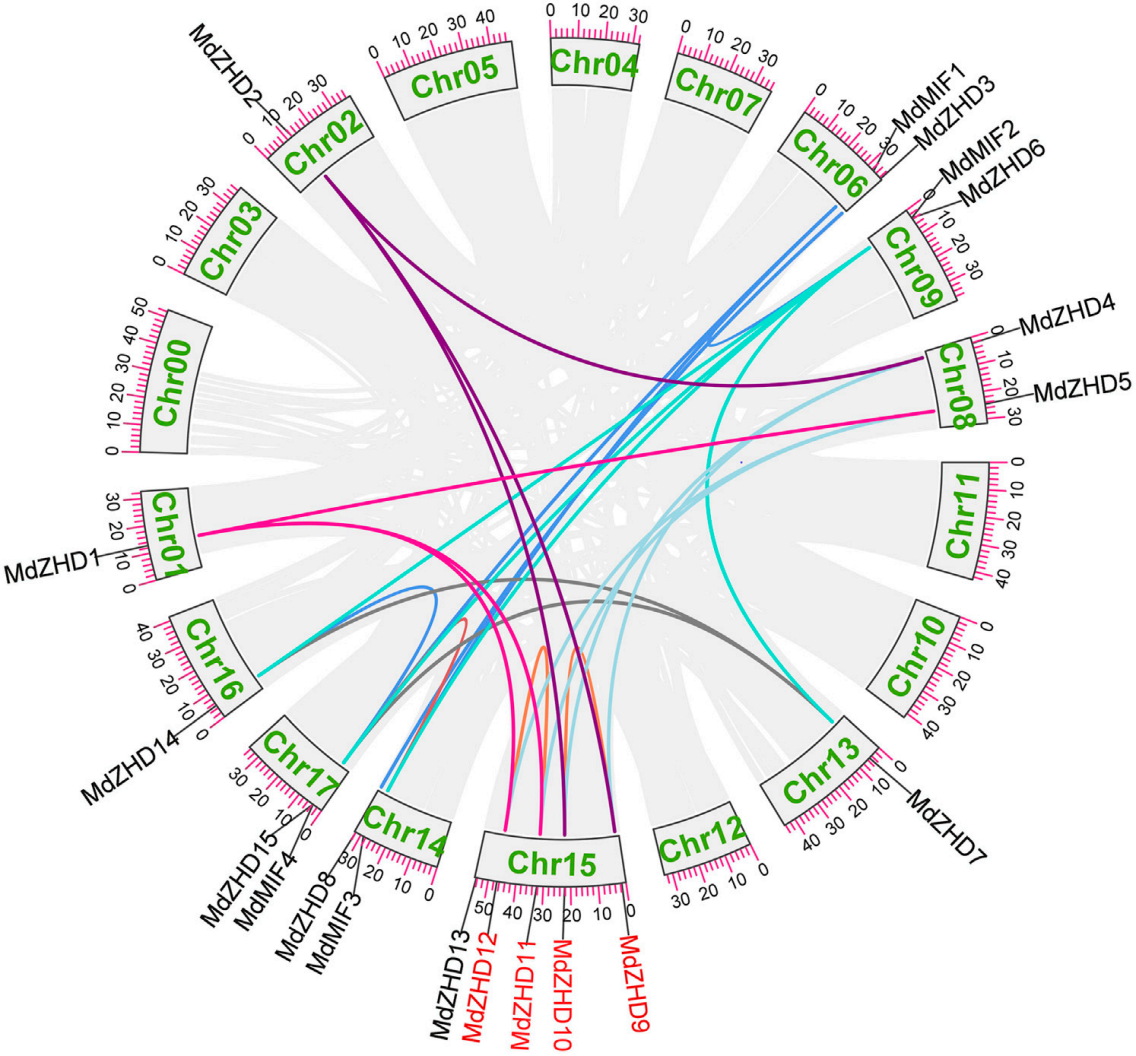
Homologous relationships and gene replications of the *ZF-HD* genes in apple. The different colored lines indicate the segmental duplicated genes, while the red gene names highlight the tandem duplicated genes.

To explore the possible relationships and potential repeated events in the MdZF-HD family, we then analyzed the collinearity of the MdZF-HD family. A total of 25 repeated events were identified, including two tandem repeated events and 23 fragments repeated events (Figure 4). The result shows that the *MdZF-HD* genes have relatively conservative and similar functions during the evolutionary process. This is also similar to the previous studies, and the genes in the ZF-HD family are highly overlapping (Wang et al., 2016). In addition, there are repeated events between *MdMIF* and *MdMIF* genes in the MIF subgroup, as well as *MdMIF* and *MdZHD* genes. The repeated events indicated that the *MIF* gene family may originate from a *ZF-HD* gene by losing the homologous domain. Accordingly, the high functional redundancy and the gene replication in this family may be due to genome replication; the functional redundancy is thus deduced to be normal in the ZHD subfamily.

### Promoter Region Analysis of the *MdZF-HD* Family Genes

**FIGURE 5:**
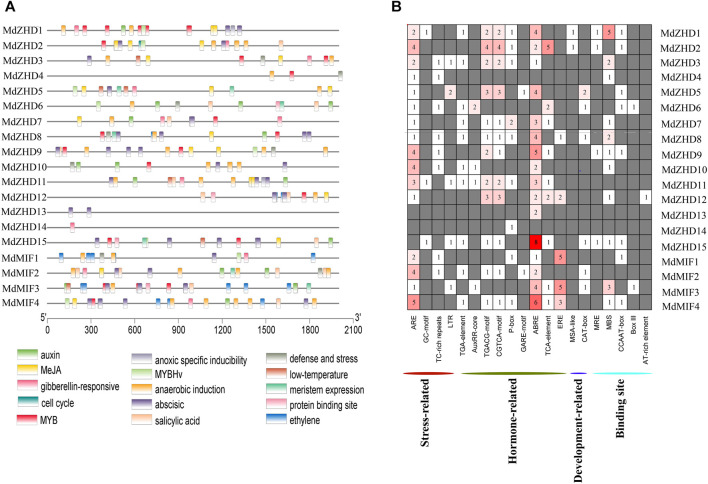
*Cis-*element analysis of the promoters of *MdZF-HD* genes. **(A)** The different colored rectangles show the different types of *cis*-elements and their positions in each *MdZF-HD* gene. **(B)** Heatmap of the numbers of *cis*-elements in the promoters of *MdZF-HD* genes.

The *cis*-elements are important regulators during plant growth and development, hormone responses as well as in response to biotic and abiotic stresses (Latchman, 1997; Yamaguchi-Shinozaki and Shinozaki, 2005). In order to explore the potential functions and the regulatory patterns of *MdZF-HD* family genes, we extracted a 2000 bp fragments upstream of the start codon (ATG) of each *MdZF-HD* gene for *cis*-elements analysis (Supplementary Table S4). The results indicated that the identified *cis*-elements can be roughly divided into four categories: stress responses (anoxic specific inducibility, anaerobic induction, defense and stress, low temperature), hormone responses (auxin, MeJA, gibberellin, abscisic acid, salicylic acid and ethylene)), the binding sites (protein binding sites, MYB, MYBHv), and development related responses (cell cycle and meristem expression) (Figure 5A).

In total, eight types of *cis*-elements responsive to different hormones including auxin response (AuxRR-core, TGA-element), MeJA response (CGTCA/TGACG-motif), gibberellin response (P-box), abscisic acid response (ABRE), salicylic acid response (TCA-element) and ethylene response elements (ERE) were found in the promoters of all *MdZF-HD* genes except *MdZHD4* (Figure 5B; Supplementary Table S5). Notably, ABRE was the most abundant *cis*-elements of these hormone responsiveness, and sixteen out of the nineteen *MdZF-HD* promoter regions contained at least one ABRE element. In addition, ERE was most distributed in the promoters of *MdMIF* genes (Figure 5B), indicating that *MdMIF* genes may be more responsive to ethylene. Furthermore, ARE was found to be distributed in almost all promoter regions of *MdZF-HD* genes, except for *MdZHD13*/*14*/*15* (Figure 5B), suggesting that the *MdZF-HD* genes may be responsive to anaerobic stress. These findings indicated that *MdZF-HD* genes may play a certain role in the regulation of gene expressions in response to hormones and abiotic stresses.

### Expression Analysis of *MdZF-HD* Genes During Apple Fruit Ripening

**FIGURE 6:**
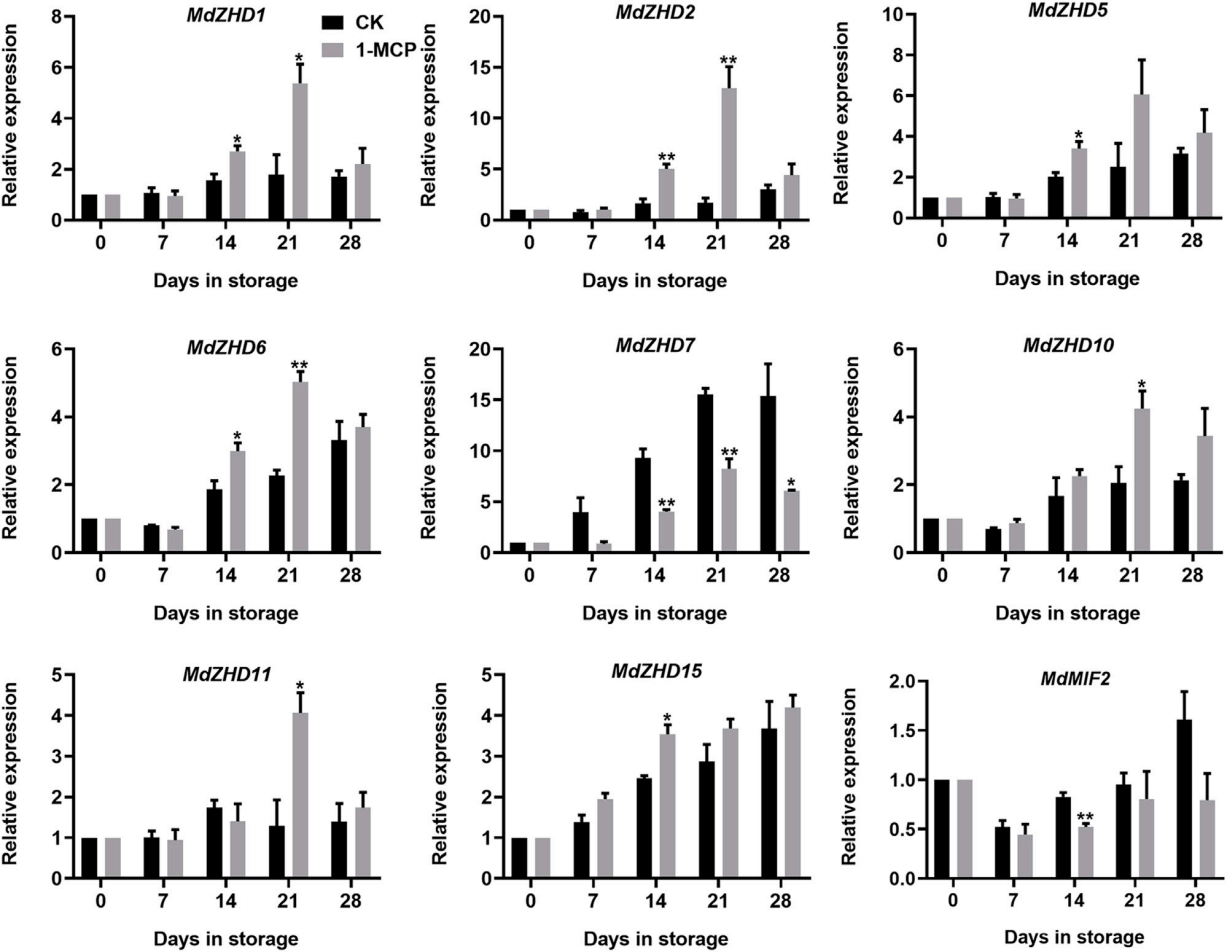
Expression patterns of *ZF-HD* genes in response to 1-MCP treatment during “Qinguan” apple fruit ripening. “Qinguan” fruit were treated with 1-MCP (1 μL L^−1^) or air (control/CK) for 24 h at 20°C. The values of day 0 fruit were set as 1. Black columns and gray columns represent the expression levels of the genes transcripts in control (CK) and 1-MCP treated fruit, respectively. Error bars represent SEs from three biological replicates (*p < 0.05; **p < 0.01).

To explore the relationship between the *ZF-HD* family genes and apple fruit ripening, the expression levels of the *MdZF-HD* genes in response to 1-MCP treatment in “Qinguan” fruit were analyzed by qRT-PCR. Based on the results, nine *MdZF-HD* genes were differentially expressed, as shown in Figure 6. Except for *MdZHD7* and *MdMIF2,* all the other genes (including *MdZHD1/2/5/6/10/11/15*) were significantly up-regulated by 1-MCP treatment and reached a peak after storage for 21 days, which showed negative association with apple fruit postharvest ripening and softening. Nevertheless, the expressions of *MdZHD7* and *MdMIF2* genes were obviously repressed by the 1-MCP treatment, which showed positive association with apple fruit postharvest ripening and softening (Figure 6).

**FIGURE 7:**
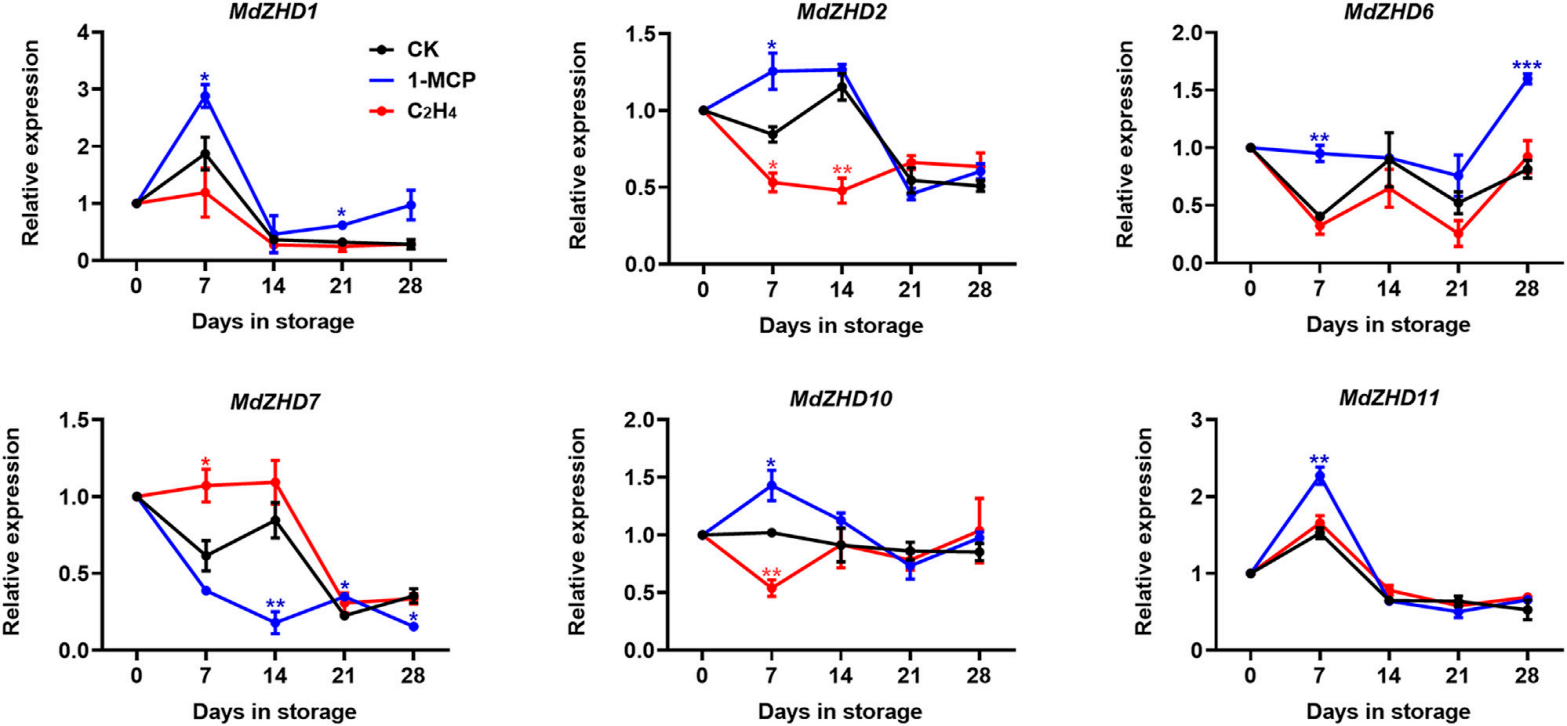
Expression patterns of *ZF-HD* genes in response to ethylene or 1-MCP treatment during “Golden Delicious” apple fruit ripening. “Golden Delicious” fruit were treated with 1-MCP (1 μL L^−1^), ethylene (C_2_H_4_, 100 μL L^−1^), and air (control/CK) for 24 h at 20°C. The values of day 0 fruit were set as 1. Black lines, blue lines and red lines represent the expression levels of the genes transcripts in control (CK), 1-MCP and ethylene treated fruit, respectively. Error bars represent SEs from three biological replicates (*p < 0.05; **p < 0.01; ***p < 0.001).

In order to verify the expression patterns of these candidate *MdZF-HD* genes in response to ethylene, six *MdZF-HD*s with higher significance levels were further analyzed in “Golden Delicious” apple fruit. Similar to the expression levels of these *MdZF-HD* genes in response to 1-MCP treatment in “Qinguan” fruit, *MdZHD1/2/6/10/11* were also significantly induced in the 1-MCP treated “Golden Delicious” apple fruit and peaked at 7 or 14 days in storage, and were mostly inhibited by ethylene treatment (Figure 7). In comparison, the expression patterns of *MdZHD7* was significantly down-regulated in the 1-MCP treated “Golden Delicious” apple fruit, and were slightly induced by ethylene treatment (Figure 7). To sum up, these six *MdZF-HD* genes could be potential candidates regulating the ethylene induced ripening and softening of postharvest apple fruit.

### Subcellular Localization of MdZF-HD Proteins

**FIGURE 8:**
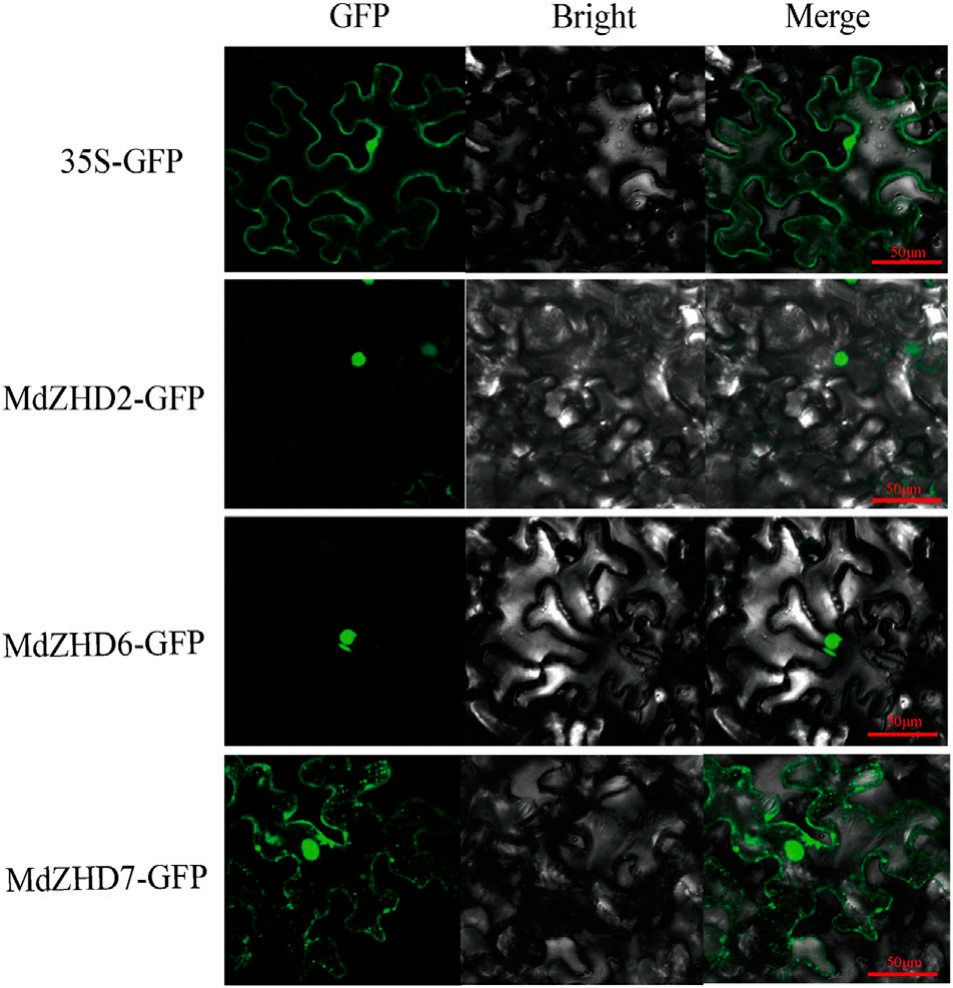
Subcellular localization of *MdZHD2/6/7*-GFP in tobacco leaves. Agrobacterium-mediated transformation was expressed in tobacco epidermal cells and the signal of green fluorescent protein (GFP) was observed by laser confocal microscope. Bars = 50 µm.

The subcellular locations of three candidate ripening related MdZF-HD proteins (MdZHD2/*6*/7) were examined in tobacco (*Nicotiana benthamiana*) leaves by using GFP tagging. The signals of the control GFP was detected in both the nucleus and cell membrane, while MdZHD2/6/7 showed strong fluorescence signals in the nucleus, with the except that MdZHD7 also gave signals in the cell membrane (Figure 8).

## Discussion

Previous studies have shown that the *ZF-HD* family genes play important roles in regulating the plant growth and development, and can enhance the resistance to stress conditions (Abdullah et al., 2018; Hu et al., 2018). To date, *ZF-HD* genes have been characterized in many plant species, such as Arabidopsis, tomato, tea tree and cucumber (Hu and Ma, 2006; Khatun et al., 2017; Lai et al., 2021; Zhou et al., 2021). Nevertheless, the evolutionary characteristics and function of the apple *ZF-HD* genes have been poorly understood, notably in regulating fruit ripening.

In present study, a comprehensive investigation of apple *ZF-HD* gene family was conducted, and nineteen *ZF-HD* genes including fifteen *ZHDs* and four *MIFs* were identified in apple (Table 1). The number of *ZF-HD* genes identified in apple is inconsistent with a previous report (Shalmani et al., 2019), on account of the different genome database we used. The number of the Z*F-HD* genes in apple was slightly higher than that of *Arabidopsis thaliana* (Hu et al., 2008), tea tree (Zhou et al., 2021) and cucumber (Lai et al., 2021). Besides, these nineteen *MdZF-HD* genes and seventeen *AtZF-HD* genes were constructed as evolutionary trees. According to the classification of Arabidopsis, the apple ZF-HD proteins can be divided into two subfamilies (ZHD and MIF), while MdZHD can be further classified into five subgroups (ZHDⅠ–ZHDV) (Figure 1), which is also consistent with the classification of ZF-HD proteins in other plants (Khatun et al., 2017; Zhou et al., 2021). After that, we analyzed the conserved motifs of MdZF-HD family proteins, and the conserved motifs between the ZHD and MIF subfamilies were significantly different, but similar conserved motifs were found among the same subfamilies (Figures 2A,B). All MdZF-HD proteins have motif1 and motif3, suggesting that ZF-HD proteins are likely to be highly conserved during evolution. *ZF-HD* genes have high plant specificity and most do not have introns (Figure 2C), indicating that ZF-HD is a relatively new family. Notably, genes containing introns (*MdZHD1*/*4/5*/*9*/*12*) are longer than those without introns in the MdZF-HD family, which is consistent with the previous reports that the number of introns is positively correlated with gene length (Zhu et al., 2020; Zhou et al., 2021). Duplication of genes can increase the number of genes (Vision et al., 2000; Blanc et al., 2003; Cannon et al., 2004; Abdullah et al., 2018). Gene replication, including fragment replication and tandem replication, is a crucial factor in the biological evolution of many plants (Kong et al., 2007). In this study, 23 fragment replications and two tandem replications were found in the chromosome distribution of *ZF-HD* genes in apple (Figure 4), indicating that the gene fragment duplications contributed to the amplification of the *ZF-HD* gene family in apple.

Promoter region analysis of the *MdZF-HD* genes identified several *cis*-elements related to phytohormones and abiotic stresses (Figure 5). Based on the previous studies, *ZF-HD* family genes were mostly found to participate in the responses to abiotic stresses (Tan and Irish, 2006; Figueiredo et al., 2012; Wang et al., 2016; Liu et al., 2021). For instance, *ZF-HD* family genes from Arabidopsis, tomato, cucumber, and also apple were shown to be up-regulated by various kinds of stress conditions, such as drought, salt, cold, heat, and phytohormones including GA and ABA (Shan et al., 2012; Zhang et al., 2015; Khatun et al., 2017; Shalmani et al., 2019; Lai et al., 2021). However, the roles of *ZF-HD* genes in regulating fruit ripening and softening have rarely been reported. In this study, nine selected *MdZF-HD* genes were differentially expressed during the postharvest ripening process of “Qinguan” apple fruit (Figure 6). Among them, *MdZHD7* and *MdMIF2*, were significantly downregulated by the 1-MCP treatment, which showed positive correlation with the postharvest ripening of apple fruit. In comparison, mRNAs from other seven genes (*MdZHD1/2/5/6/10/11/15*), especially *MdZHD1/2/6*, displayed increase in abundance in response to 1-MCP treatment during the postharvest storage of “Qinguan” apple fruit, which showed negative association with apple fruit ripening. In addition, six candidate *MdZF-HD* genes with higher significance levels were further analyzed in another cultivar “Golden Delicious,” and showed similar expression patterns in response to the1-MCP treatment. Of the six *MdZF-HD* genes, five genes (*MdZHD1/2/6/10/11*) were repressed and one gene (*MdZHD7*) was slightly induced in response to ethylene treatment (Figure 7), implying that they may be involved in the regulation of the ethylene induced ripening of postharvest apple fruit. The detailed regulatory mechanisms remain to be further investigated. Furthermore, the subcellular localizations of three selected *ZHDs* (*MdZHD2*/*6*/*7*) in nucleus were consistent with the prediction (Table 1; Figure 8), and the MdZHD7 protein was also located in the cell membrane. The results showed that these three genes can be located in the nucleus, indicating that they may have functions as transcription factors. Similar to this phenomenon, some transcription factors are not only located in the nucleus. For instance, the TaMIF4-5D in *Triticum aestivum* was located in the nucleus and cell membrane (Niu et al., 2021); the CitNAC62 in citrus was not located in the nucleus and its subcellular location was within plastids (Li T. et al., 2017).

## Conclusion

Nineteen *ZF-HD* family genes were newly identified in apple, and their phylogenetic relationships, gene structures, conserved motifs, subcellular localizations, as well as their expression patterns in response to ethylene or 1-MCP treatment during the postharvest storage of apple fruit were analyzed. Besides, the expressions of several *MdZF-HD* genes in apple fruit of two cultivars were obviously altered in response to ethylene or 1-MCP treatment. Our findings may supply valuable clues for identifying the potential roles of *MdZF-HD* genes in regulating the fruit ripening.

## Data Availability

The original contributions presented in the study are included in the article/Supplementary Material, further inquiries can be directed to the corresponding author.
