# Supplementary figures and images for: Genome-Wide Investigation of the Zinc Finger-Homeodomain Family Genes Reveals Potential Roles in Apple Fruit Ripening
Source: Front Genet. 2022 Jan 17;12:783482. doi: 10.3389/fgene.2021.783482 (PMC8802310; doi:10.3389/fgene.2021.783482)

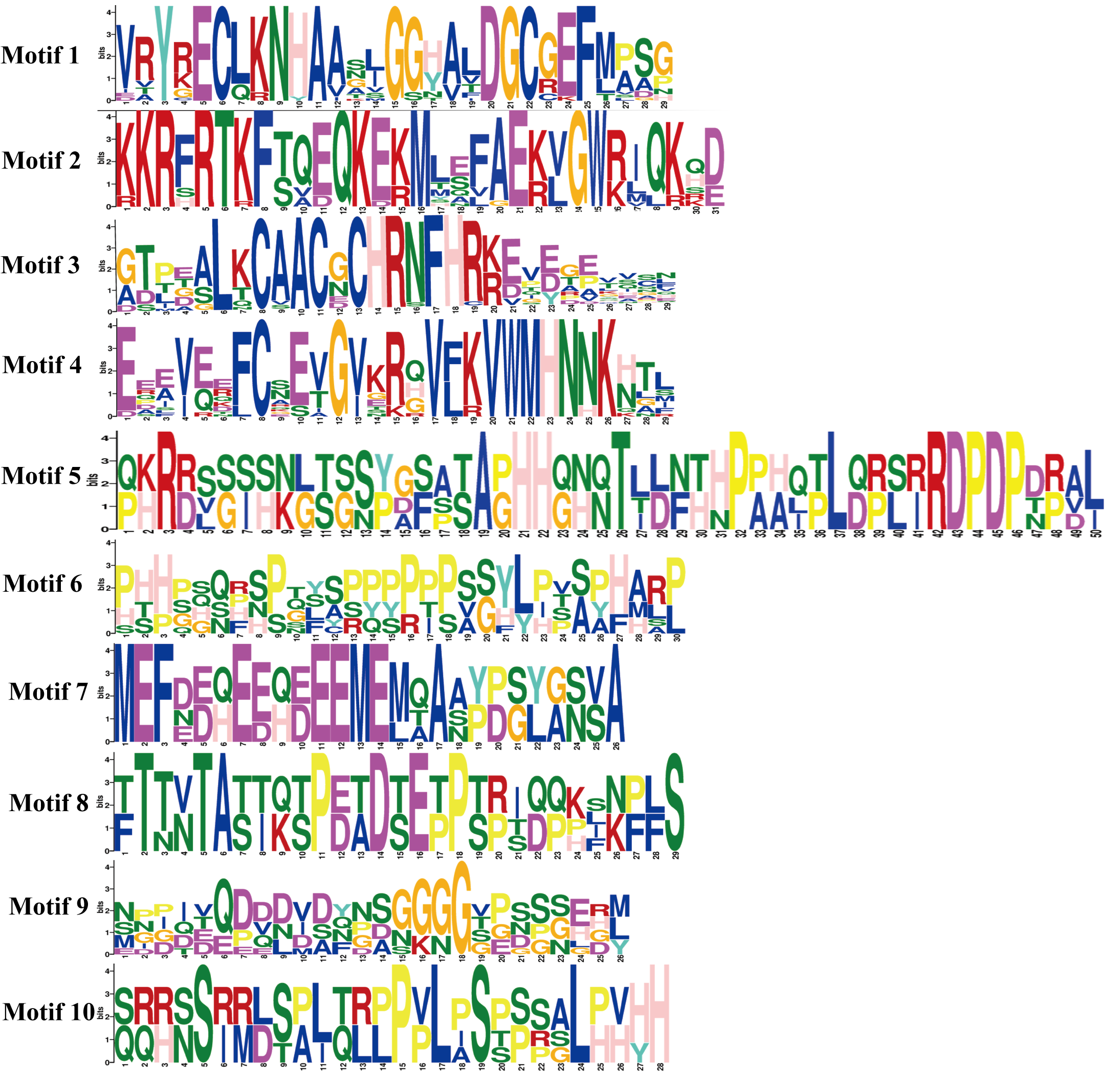

Supplement: Supplementary file 2 [file Image1.TIF]
