# Supplementary material for: Genome-Wide Investigation of the Zinc Finger-Homeodomain Family Genes Reveals Potential Roles in Apple Fruit Ripening
Source: Front Genet. 2022 Jan 17;12:783482. doi: 10.3389/fgene.2021.783482 (PMC8802310; doi:10.3389/fgene.2021.783482)
Supplement: Supplementary file 3 [file DataSheet1.docx]

Supplementary Material

# Supplementary Figures

**
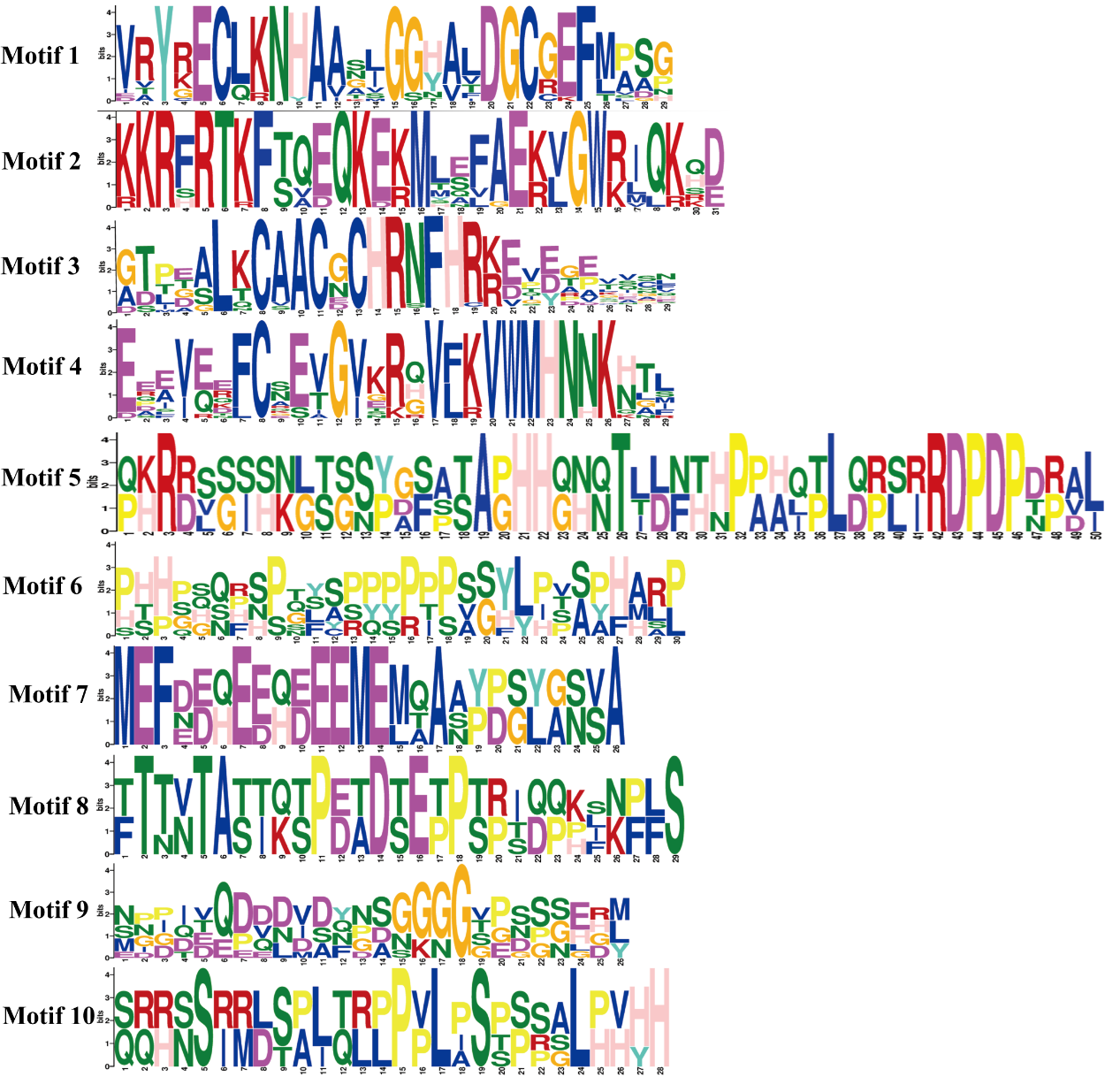
**

**Supplementary Figure S1.** The motif logos of apple *ZF-HD* genes*.*

**
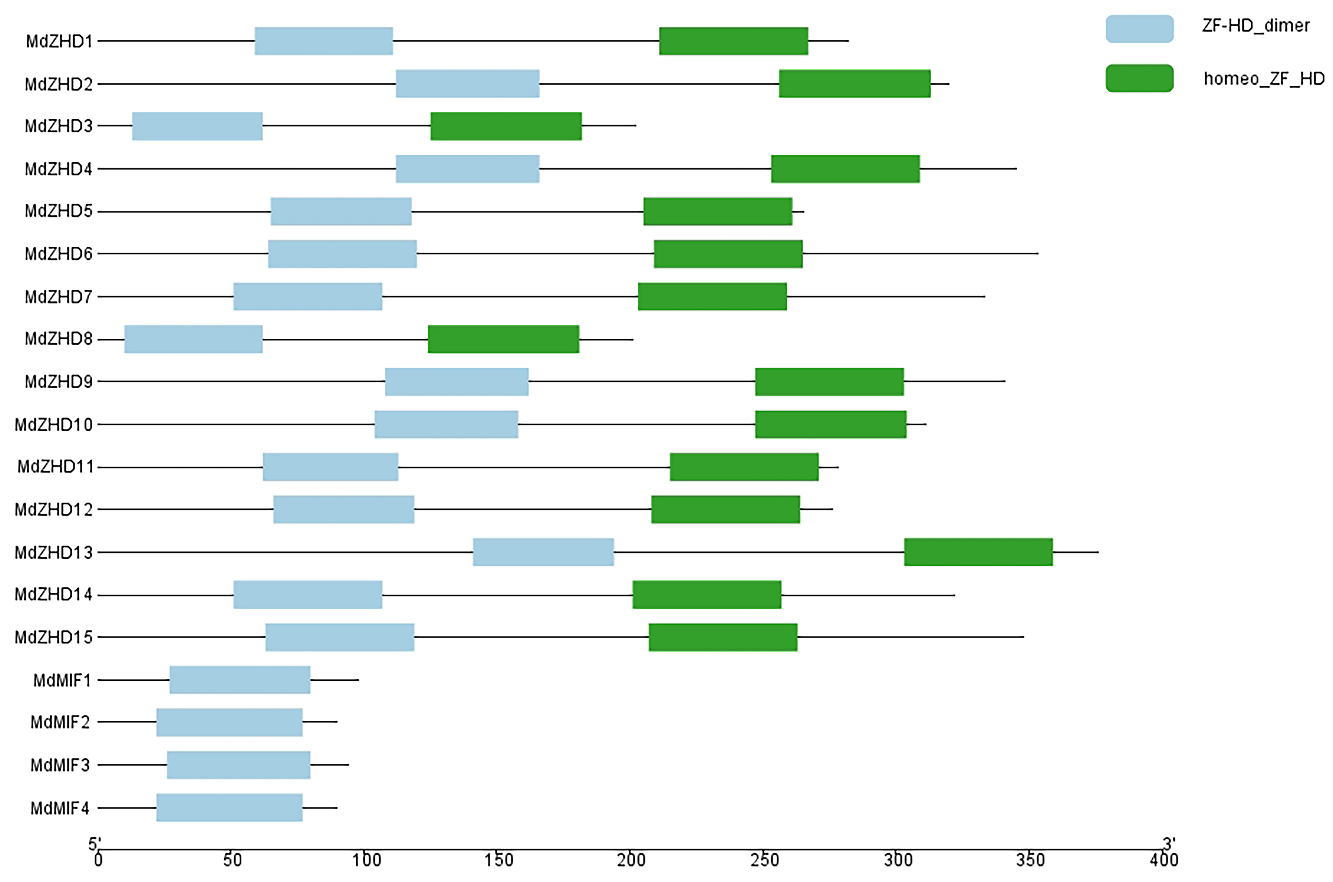
**

**Supplementary Figure S2.** The visual conserved domains of MdZF-HD proteins based on the predicted result of CDD.

# Supplementary Tables

**Supplementary Table S1.** List of primers used for the qRT-PCR.

| **Gene Name** | **Primer sequence (5'-3')-FP** | **Primer sequence (5'-3')-RP** |
| --- | --- | --- |
| *MdActin* | GGATTTGCTGGTGATGATGCT | AGTTGCTCACTATGCCGTGC |
| *MdZHD1* | ACTCGCTTGGAAACTCCGCC | TCCCCACCGCGTGGTTCTTT |
| *MdZHD2* | TGGGGTGGTTGCGGAGTCAT | TCCGCCACCTCCGTCATCTTCT |
| *MdZHD5* | TGCAGAGAGTTCCAGGCCCA | ACGGCGTGGTTCTTGAGGCA |
| *MdZHD6* | TCCTTCAGCAACGGCGTCCT | AGCGGGAGACGGCATGAACT |
| *MdZHD7* | ATTGGGCAGCGGAAACGGCA | ACGACGAAGACGACGACCCA |
| *MdZHD10* | TTTGGCAGCAGTGGTGGGGT | AACTTCTCCGCCACCTCCGT |
| *MdZHD11* | AGCCGGAGGCCGAGTCAAAA | ATCCCCACCGCGTGGTTCTT |
| *MdZHD15* | AGCAGACCCACCCCCACAAT | TGTTGGCACCACCGTCAGCA |
| *MdMIF2* | GTCGGAGGCTACGCTGTTGA | AGGCGAAGAGCACTCGCAGA |

**Supplementary Table S2.** List of primers used for the subcellular localization.

| **Gene Name** | **Primer sequence (5'-3')-F** | **Primer sequence (5'-3')-R** |
| --- | --- | --- |
| *MdZHD2-GFP* | gtggatccaaagaattc ATGGAACAGAGAGGGCGAGA | ctcctttacccatgaattcCATTTGCTTCTTCTTCA |
| *MdZHD6-GFP* | gtggatccaaagaattcATGGATATAACCCCGTCCAT | ctcctttacccatgaattcAGAAGAAGAAGACGACC |
| *MdZHD7-GFP* | gtggatccaaagaattcATGAACTTTACCACCGTCAC | ctcctttacccatgaattcCGACGAAGACGACGACC |

**Supplementary Table S3.** The deduced amino acid sequences of the *ZF-HD* genes in apple and Arabidopsis used for the phylogenetic analysis.

| **Gene Name** | **Protein sequences** |
| --- | --- |
| MdZHD1 | MEFNDQEDHEEEMEMTANYDSLGNSAGGRVKMSSSGPDGLALAAAAAAATQQQQQPRKVVRYRECLKNHAVGIGGHALDGCCKFLAAGPEGTLDALKCAACNCHRNFHRKEGDPPGHGLGVITDACGQLVPHHGQQHHPQFSPYYRTPAGFLHVAAHHRLLALPSTSGGGGTHSGGQEQDDDVSNPSGGGGGGSGGLGMGMGMHGTASSGKKRFRTKFSQEQKERMLSLAERLGWRIQKQDEPAVQQFCNETGVNRHVLKVWMHNNKHTLAIWRRICLKRFN |
| MdZHD2 | MEQRGRDNKVIGRMPSTLTYTPPPPHRDSSSSKLSSSPAFSSAGHHGNNTILFTHPPHQTLDRSRRDPDPNPDLVPSPLVVTPSATTTIASGTNFKAPAAPPPPPPSAAASRIRYRECLKNYAANSGGHVLDGCGEFMPSGDEDTPGALKCAACECHRNFHRKEIDGEYVSNNYYVINHQQHNSRRDSALTRLPVLASSPRGLPVYHSMAGGPSPPMMMTFGGGGGVVAESSSEDLNMNNKLFRGANYAEEAQGSKKRFRTKFSQEQKEKMTEVAEKLGWRIQKHDEQEVQKLCSEVGIKRQVFKVWMHNNKKAMKKKQM |
| MdZHD3 | MEGDHDPNTNDVYRECLRNHAASLGSYATDGCGEFTVDHASPGGLQCAACGCHRNFHRRVTYAATSSQAAGGGRSGHLQHHVIMSCSSRGRDPAENIITTQDQLIDYNAGGGGSPDSGERMSSEKKRFRTKFTAEQKEKMLAFAEKLGWKLLRKDLEDEIETFCRSVGVTRQVFKVWMHNHKNLSSSSTSASTGNASSLTTQ |
| MdZHD4 | MDFRAQDNEMRTSGSLSYAHLNTKESSPSPAAAADHQQKRRVGIHNGTGNYGSATAPHHQHQTLDNHHPAAIPLQPLIRDPDPDRALSGTPVAPHEAGVGGGGGGPKSLAKVVRYRECLKNHAANIGGNVFDGCGEFMPSGQEGTMEALKCAACDCHRNFHRKEVDGETAAFSPGSRRSSIMLSPLQLPPPLPSPSSALHHHHHHHHHQKYAMPPIVQPVNVAFGSSGGGTESSSEDLNAFEGGTVPPFALSKKRFRTKFTVEQKERMMEFGEKVGWRIQKQDEEEVERFCAEVGVKRQVLRVWMHNNKNTIKKQGDVTATTNAIALSFKNVEGEGGEGGAAAED |
| MdZHD5 | MEFDEHEEQDEEMEMQAAPPGYASVAESSRPKMGPAGEETVSSARKRGTAAAPNSTTTTTLATMVRYRECLKNHAVTIGGHALDGCGEFLSAGDEGTIDALKCAACNCHRNFHRKESEGELIRHHQGAGGAHHHHHQHQFSSGYCRPPPPPSGYLITSPHARPTLALPAASIGGRGGSHSREEGEDVSNPSSSGGGGGGGFGMSKKRHRTKFTQEQKEKMLEFAEKVGWRIQKHDEAAIEEFCGETGVKRHVFKVWMHNNKHTLD |
| MdZHD6 | MDITPSITTTTNNTASTKSPEADSETPTRIQQPLKPLSFSNGVLKRHNPTHHLHHQNIPITPVVVTYKECLKNHAAALGGHALDGCGEFMPSPAANLADPTSLKCAACGCHRNFHRRDPEDPVQPNTPAATTHVIEYQPHHRHHPPPPTHPGNRSPSSASPPPISSSYYPSAPHMLLALSTAHENALAGANNNNAVAMPVMSRSPNARKRFRTKFTQDQKEKMYQFAERVGWKMQKRDEEIVREFCNEAGVEKGVLKVWMHNNKNTFSKRDVLNGGAGGRAGSLSRPSFLLEHSHHHNNGTNGNGTNGNGNNNDDDEEEDDDQNDNKNGVPNPNHHYQGADGGGNNGSSSSS |
| MdZHD7 | MNFTTVTATIQTPDTDTEPPSPSDPKSNFFSSRSLSFTNGAFQPQSTPTMVVAYKECLKNHAASLGGHALDGCGEFMPSPSSNPADPTSLKCAACGCHRNFHRRDQYRPKSNVIRNPHRLLPAPKPAHYNHSSSPSPSSSPNPTLSPQSPPPVSHLPPSYFASPPQMLLALSSGFSGPSDEHPHQHQLNPTAVKTEKYPGEKKRSRTRFSQEQKEKMLSFAEKVGWRLQKSEERLVEDFCSEVGIGRGVFKVWMHNNKHGRRRLERLGSGNGSLGDGGNMNKNVSEINGDGERLGFDSMNAHIASYNVNANPLNEGSRASFHLSTNGSSSSS |
| MdZHD8 | MEGDHDPNTNEVYRECLRNHAASLGSYATDGCGEFTLDHASPGGLQCVACGCHRNFHRRVTYAATSSQAAGGGRSGHQQHHVIMSCSSRGRDPAENIITQDQLIDYNAGGGGSPDSGERMSSGKKRFRTKFTAEQKEKMLAFAEKLGWKLLRKDLEDEIERFCKSVGVSRQVFKVWMHNHKNLSSSSTSASTGNASSLTTQ |
| MdZHD9 | MRTSGSLSYAHLSAKESSPSSAAAAPAADHQQKRRLGIHNGTGSYGSATAPHHQHQTLDNHNPAALPLQPLIRDPDPDRALSGTPVAPHGARLSGSGGGGGPKSLGKVVRYRECLKNHAANIGGNVFDGCGEFMPSGEEGTLEALKCAACDCHRNFHRKEVDGETAAFSPGSRRSSIMLTPLQLPPPLPSPSSALHHHHHHHQKYSMPPIVQPVNMAFGSGGGGTESSSEDLNAFEGGAVPPFALSKKRFRTKFTVEQKERMMEFAERVGWRIQKQDEEEVERFCAEVGVKRQVLRVWMHNNKNTIKKQGDVTATTNAIATGLSFDNVEGEGGEGAAAAED |
| MdZHD10 | MELRGQDKVIGRMPSTLTYTPPPHRDSSSSKLSSSPDFPSAGHHGNNTTLFTHPPHQTLDRSRRDPDPTPVIVTPSAATTIASGSNFKAPPAPPQPQPSTAASRVRYRECLKNHAASSGGHVLDGCGEFMPGGDEDTPGSLKCAACECHRNFHRKEIDGDHVSNNYYVINQQHNSRRDSAITRLPVLISTPPSLPVHHSAARGPSPAMMMTFGSSGGVAAESSSEDLNMNNNLFGGANYAADAQGSKKRFRTKFSQEQKEKMTEVAEKLGWRIQKHDEQEVEKLCSEVGIKRQVFKVWMHNNKQAMKKKQM |
| MdZHD11 | MEFEDQEEHEEEMEMAASYDSLGNSAGGRVKMSSSGPDGLALAAAAAAAAASDQQQQPRKAVRYRECLKNHAVGIGGHALDGCCEFLAAGPDGTLDALKCAACNCHRNFHRKEGDPAGHGLGVITDAYGQLVPHHGQQHHPQFSPYYRTPAGYLHVAAHHRPLALPSTSGGGGTYSGGQEQDDDVSNPSGGGGGGGGSGGLGMGMGMHGTASSGKKRFRTKFSQEQKERMLSLAERLGWRIQKQDEPAVQQFCNETGVKRHVLKVWMHNNKHTLGKKP |
| MdZHD12 | MEFDEHEEQDEEMELQAAPPGYASVAASSRPKLGPTGEGAASIVRKRGTAATPNSTTTTTAVSTMVRYRECLKNHAIGIGGHALDGCGEFLAAGDEGTLDALKCAACNCHRNFHRKEPEAELIHHQQGAGGSHHLHHHHHPHQFSSGYYRPPPPPSGYLITSPHARPTLALPAASGGGGGSHSREEEEDVSNPSSSGGGGGGGFGMSKKRHRTKFTQEQKEKMLEFAEKVGWRIQKHEEAAIEEFCEETGVKRHVFKVWMHNNKHTLVFTASSLPV |
| MdZHD13 | MDQLSSQEGEIPITIPVPIPIPINTSFGSGGHGHLIHHHQQQRPHDPTAAATARAAVPHNNNKNNHSNFLPSSTVVPQNQTPHNTNGSNPMPTSLDHSHEEEEEQEEEHHDHVNNANANVVPYKYNTYNNKKQAVVGHNGVRYKECLKNHAAAMGGTATDGCGEFMPSGEEGTIEALNCSACNCHRNFHRKEVEGEQQQLSSCDYNFHHTINRAAGVGSRKFLLSHEGGGGGRHKSLLAPPQYPHQHQMIMSNYNMGMGMIEMVGSIPSDQSDEQEDHHHHRHHGGGGGGTVGSRPAVGHVAKKRFRTKFTQEQKEKMLNFAEKVGWKIQKQEDSFVQNFCQEIGVKRRVLKVWMHNNKHNLANKINPPPPPPPAP |
| MdZHD14 | MDFTTVTATIQTPDTDTEPPSPTDPKFNFFSGRSLSFTSGAFQPQPKRTRVVAYKECLKNHAASLGGHALDGCGEFMPSPSSNPADPTSLKCAACGCHRNFHCRDQYRPKNNVIRNRLLPAPKTAHNNHSSSSSPSSSPNPTLSPQSPPPVSHLPPSYFAAPPQMLLALSSGFSGPPDEHPHQHQLNPTAVMKTEKYPGEKKRSRTKFSQEQKEKMSSFAEKVGWRVQKSDERSVEDFCSEVGIGRGVFKVWMHNNKHGLRRRLERSAGVGGGGNMIINSNVSEINGEGERHGFDSMNAHIADNNVNANPLHLSNNGSSSSS |
| MdZHD15 | MDITPPITTTTNTASTKSPEADSETPTRIQQHIKPLSFSNGVLKRHNPTHHLHHHNIPITPVVVTYKECLKNHAATLGGHALDGCGEFMPSPTANSADPTSLKCAACGCHRNFHRRDPEDPVQQNTPAATTHVIEYQPHHRHHPPPPTHPGNRSPNSASPPPISSSYYPSAPHMLLALSTAHENALVGLNNHAAVMPPIVSASPNARKRFRTKFTQDQKDKMHQFAERVGWKMQKREEEIVQEFCNEVGVEKGVLKVWMHNNKNTFTKRDVLNGGGAGGGLGRPNFLLQQTHPHNGTNGNGNGNGNNNDDDDEDDDDDQDDNKNGVPNPNHHYQGADGGANNGSSSSS |
| MdMIF1 | MKKRQVVVKRDRSERNSSTSSSMVRIVRYGECQKNHAAKLGGYAIDGCREFMASGEEGTTEALTCAACGCHRSFHRKEVETEVVCEYSPPSSYRKWCT |
| MdMIF2 | MRKRQVVLRRTEQASAASSFTVVRYGECQKNHAAGVGGYAVDGCREFMASNGEEGTTAALTCAACGCHRNFHRREVETVCECSSPSSNGA |
| MdMIF3 | MKKRQVVVKRDRSGRSSSTSSSMVRTVRYCECQKNHAANLGGYAVDGCREFMASGEDGTTEALTCAACGCHRNFHRREVETEVVCEYSPPNSYR |
| MdMIF4 | MRKRLVVLRRTEEASAASSFTVVRYGECQKNHAAAVGGYAVDGCREFMASNGEEGTTAALTCAACGCHRNFHRREVETVSECFSPSSNGA |
| AtZHD1 | MEFEDNNNNNDEEQEEDMNLHEEEEDDDAVYDSPPLSRVLPKASTESHETTGTTSTGGGGGFMVVHGGGGSRFRFRECLKNQAVNIGGHAVDGCGEFMPAGIEGTIDALKCAACGCHRNFHRKELPYFHHAPPQHQPPPPPPGFYRLPAPVSYRPPPSQAPPLQLALPPPQRERSEDPMETSSAEAGGGIRKRHRTKFTAEQKERMLALAERIGWRIQRQDDEVIQRFCQETGVPRQVLKVWLHNNKHTLGKSPSPLHHHQAPPPPPPQSSFHHEQDQP |
| AtZHD2 | MNFEDQEEDMEMSGVNPPCGYDSLSGEGATSSGGGGVGRSKGVGAKIRYRECLKNHAVNIGGHAVDGCCEFMPSGEDGTLDALKCAACGCHRNFHRKETESIGGRAHRVPTYYNRPPQPHQPPGYLHLTSPAAPYRPPAASGDEEDTSNPSSSGGTTKRFRTKFTAEQKEKMLAFAERLGWRIQKHDDVAVEQFCAETGVRRQVLKIWMHNNKNSLGKKP |
| AtZHD3 | MEIASQEDPIPINTSYGNSGGGHGNMNHHHHANSAPSSLNITTSNPLLVSSNSNGLGKNHDHSHHHHVGYNIMVTNIKKEKPVVIKYKECLKNHAATMGGNAIDGCGEFMPSGEEGSIEALTCSVCNCHRNFHRRETEGEEKTFFSPYLNHHQPPPQQRKLMFHHKMIKSPLPQQMIMPIGVTTAGSNSESEDLMEEEGGGSLTFRQPPPPPSPYSYGHNQKKRFRTKFTQEQKEKMISFAERVGWKIQRQEESVVQQLCQEIGIRRRVLKVWMHNNKQNLSKKSNNVSNNVDLSAGNNDITENLASTNP |
| AtZHD4 | MEIASQEDHDMPIPLNTTFGGGGSHGHMIHHHDHHAANSAPPTHNNNNTTQPPPMPLHGNGHGNNYDHHHHQDPHHVGYNAIIKKPMIKYKECLKNHAAAMGGNATDGCGEFMPSGEDGSIEALTCSACNCHRNFHRKEVEGELAATAMSPYHQHPPHRKLMLNHQKIRSAMPHQMIMPIGVSNYRYMHNNSESEDFMEEDGVTTASRSLPNLPYNQKKRFRTKFTPEQKEKMLSFAEKVGWKIQRQEDCVVQRFCEEIGVKRRVLKVWMHNNKIHFSKKNNINLEDNDNEKINNLNNVDLSGNNDMTKIVP |
| AtZHD5 | MDMRSHEMIERRREDNGNNNGGVVISNIISTNIDDNCNGNNNNTRVSCNSQTLDHHQSKSPSSFSISAAAKPTVRYRECLKNHAASVGGSVHDGCGEFMPSGEEGTIEALRCAACDCHRNFHRKEMDGVGSSDLISHHRHHHYHHNQYGGGGGRRPPPPNMMLNPLMLPPPPNYQPIHHHKYGMSPPGGGGMVTPMSVAYGGGGGGAESSSEDLNLYGQSSGEGAGAAAGQMAFSMSSSKKRFRTKFTTDQKERMMDFAEKLGWRMNKQDEEELKRFCGEIGVKRQVFKVWMHNNKNNAKKPPTPTTTL |
| AtZHD6 | MEVREKKDEKMEMTRRKSSALDHHRLPPYTYSQTANKEKPTTKRNGSDPDPDPDLDTNPISISHAPRSYARPQTTSPGKARYRECQKNHAASSGGHVVDGCGEFMSSGEEGTVESLLCAACDCHRSFHRKEIDGLFVVNFNSFGHSQRPLGSRHVSPIMMSFGGGGGCAAESSTEDLNKFHQSFSGYGVDQFHHYQPKKRFRTKFNEEQKEKMMEFAEKIGWRMTKLEDDEVNRFCREIKVKRQVFKVWMHNNKQAAKKKDL |
| AtZHD7 | MELGGKCNAITTTTMISTEVKPHTDPEPEAKPESDPSMALFPIKKENQKPKTRVDQGAKYRECQKNHAASTGGHVVDGCCEFMAGGEEGTLGALKCAACNCHRSFHRKEVYGHRNSKQDHQLMITPAFYSSNSSYKPRVMHPTGEIGRRTSSSSEDMKKILSHRNQNVDGKSLMMMMMRKKKRVRTKINEEQKEKMKEFAERLGWRMQKKDEEEIDKFCRMVNLRRQVFKVWMHNNKQAMKRNNSNISE |
| AtZHD8 | MDVIATTTTIVSDLDSRQPEIEAPIRIQPAKPISFSNGKRCHHHHLASEAVAVATYKECLKNHAAGIGGHALDGCGEFMPSPSFNSNDPASLTCAACGCHRNFHRREEDPSSLSAIVPAIEFRPHNRHQLPPPPPPHLAGIRSPDDDDSASPPPISSSYMLLALSGGRGGANTAVPMSRKRFRTKFSQYQKEKMFEFSERVGWRMPKADDVVVKEFCREIGVDKSVFKVWMHNNKISGRSGARRANGGVVVGGVGDSRQSVVPTNGSFSST |
| AtZHD9 | MLEVRSMDMTPKSPEPESETPTRIQPAKPISFSNGIIKRHHHHHHNNNKVTYKECLKNHAAAIGGHALDGCGEFMPSPSSTPSDPTSLKCAACGCHRNFHRRETDDSSAVPPPSLLPSSTTTAAIEYQPHHRHHPPPPLAPPLPRSPNSSSPPPISSSYMLLALSGNNKTAPFSDLNFAAAANHLSATPGSRKRFRTKFSSNQKEKMHEFADRIGWKIQKRDEDEVRDFCREIGVDKGVLKVWMHNNKNSFKFSGGGATTVQRNDNGIGGENSNDDGVRGLANDGDGGGGRFESDSGGADGGGNVNASSSSS |
| AtZHD10 | MMDMTPTITTTTTPTPKSPEPESETPTRIQPAKPISFSNGIIKRHHHHHHPLLFTYKECLKNHAAALGGHALDGCGEFMPSPSSISSDPTSLKCAACGCHRNFHRRDPDNNNDSSQIPPPPSTAVEYQPHHRHHPPPPPPPPPPRSPNSASPPPISSSYMLLSLSGTNNNNNNLASFSDLNFSAGNNHHHHHQHTLHGSRKRFRTKFSQFQKEKMHEFAERVGWKMQKRDEDDVRDFCRQIGVDKSVLKVWMHNNKNTFNRRDIAGNEIRQIDNGGGNHTPILAGEINNHNNGHHGVGGGGELHQSVSSGGGGGGFDSDSGGANGGNVNGSSSS |
| AtZHD11 | MDLSSKPQQQLLNSLPIAGELTVTGEMGVCYKECLKNHAANLGGHALDGCGEFMPSPTATSTDPSSLRCAACGCHRNFHRRDPSENLNFLTAPPISSPSGTESPPSRHVSSPVPCSYYTSAPPHHVILSLSSGFPGPSDQDPTVVRSENSSRGAMRKRTRTKFTPEQKIKMRAFAEKAGWKINGCDEKSVREFCNEVGIERGVLKVWMHNNKYSLLNGKIREIEHGLCLNTHSNDGDGSSSS |
| AtZHD12 | MVVLYNECLKNHAVSLGGHALDGCGEFTPKSTTILTDPPSLRCDACGCHRNFHRRSPSDGFSQHRSPPSPLQLQPLAPVPNLLLSLSSGFFGPSDQEVKNKFTVERDVRKTAMIKKHKRTKFTAEQKVKMRGFAERAGWKINGWDEKWVREFCSEVGIERKVLKVWIHNNKYFNNGRSRDTTSSMSLNLKL |
| AtZHD13 | MDEIKPKKEENSKRRRNVKPICRETGDHVHYLPTCKTKPKPTRTHHAPPPILDSIFKVTHKPHYYECRKNHAADIGTTAYDGCGEFVSSTGEEDSLNCAACGCHRNFHREELIPENGGVTETVLEVLKISSCQFRRIFCSPYGGGKSEGKKKKKEKESYGGDPIIKDRFGGAEEEEGIVKRLKTKFTAEQTEKMRDYAEKLRWKVRPERQEEVEEFCVEIGVNRKNFRIWMNNHKDKIIIDE |
| AtZHD14 | MQSTCVYRECMRNHAAKLGSYAIDGCREYSQPSTGDLCVACGCHRSYHRRIDVISSPQINHTRFPFTSLRRVKQLARLKWKTAEERNEEEEDDTEETSTEEKMTVQRRRKSKFTAEQREAMKDYAAKLGWTLKDKRALREEIRVFCEGIGVTRYHFKTWVNNNKKFYH |
| AtMIF1 | MMKKRQMVIKQRSRNSNTSSSWTTTSSSSSSSEISNVRYVECQKNHAANIGGYAVDGCREFMAAGVEGTVDALRCAACGCHRNFHRKEVDTEVVCEYSPPNA |
| AtMIF2 | MRKRQVVLRRASPEEPSRSSSTASSLTVRTVRYGECQKNHAAAVGGYAVDGCREFMASRGEEGTVAALTCAACGCHRSFHRREIETEVVCDCNSPPSTGN |
| AtMIF3 | MKKRQVVIKQRKSSYTMTSSSSNVRYVECQKNHAANIGGYAVDGCREFMASGGDDALTCAACGCHRNFHRREVDTEVVCEYSPPNANN |

**Supplementary Table S4.** The promoter sequences of apple *ZF-HD* genes.

| **Gene Name** | **Sequences** |
| --- | --- |
| *MdZHD1* | tgccaggaaggtaaaatttgtaaactaaatgatgtgtcatcaataggaataaatatgtttattaacgctcaagtaacaaccaatcatcaatttttatgttttttagttttcaaaattttatctacaagtttagttttcctaacattagccaaatatgaatgtcctccttttgaaaagaccaacatcatttgtatgcagaaattggttggtaaggccatctccaactgatagatggccaggggctcgttttagccctctggccctccaaaatattaatattttaatgaacagtacattgttatatttgcctccgtctccaccgagggccaaagggccagatgactcgttttagccttgtcacaaaaaacaatctccaactgagggccaaagggcaaacataatttattgtttaaatttaaaaactataacaacttaaattcaaatccaacaacttaaatttaaaaactacaactatatttaaaaacgacgaattaaatttaaaaactacaaattaaatttaaaggaatggtgaatgaatggtttatgtatttataggaaaaacttagaatttttatgaattaaaaaaaaggcccaaaaacctaaaaaaaaaaaattgaatccaacggtaatagcgctagctagcagttggattcaaattttagtttaagcattcctatcggttataactgacaggaatacatttatattaataatataaatctatttctgtcggttatcaccgacaagattggtgtttgttctggccagcccttcagccctttggccctttagattccatgaggccctctcatattccactagccctctggcctagccttcggttggagattatttttgagttattttcgccctttagacccttcggttggagatggcctaataagtcctatggaaccaaatgcgtgcagttgcagttgcaccccaaacaacacaccaaaaccgaagagttgtattgcagtgcagtatagtgttttttgttacaacaaaaacccttccaatatttgctctcttttctcagacagaaacgcatgaaacgcatgaaactattccaatattaaatgcaaaattttgacgccaaatgagcgtcacgcatgtcggaaacgagtgcccgtaccccagctgccttaaatgccgacaatttattttttcctatgtaagccgtgtcctgacacgtccgatatatacaacacaaacgcacctctctcccccgtacgcgacacaccacgcgccacaccttgcttcctgctggaacgtgtccaacaccacctcagcgcgtgcgacactttatcttttgctttttcttttttcatttccatatgtttgtttttatttatagagtatattgcctcattcggacttctaaaaatgatgaaaaaagattttttagaaacaactttagtaaaaatgtaaataaatttaaaaaaacatttgaagtgcacttgtatcaagcacaagttatgttgtttttgcaaaaaaactttaatcactttcgccattcaaaaatatttttttataaatcgctttcaattattttgaattatttttaaacgagttctatctgaattagagtagtctgtctgacaatcctccacagctctctgaacaagcgcggtgaaaaaacggagggtgaaagggaaagtgaccggttcaaccgggagtagtcggttgggattgagaaaatgggaaagtatatgaaggacttacaggaagacaagctattttgcttgcagggagagaggtggtgaggggagtgagacaatacacagaatttacacctgacaacacacacacacacactctctctctctctctctctctctctctctctctctctctctctctctctctctctctctctctctctcctttatcttcttctttctgggttgatactcttttattacttagtggggtttttctgtttttctctgattttaatcaattgattaattgg |
| *MdZHD2* | atacagcatcaaattacaaagggaaagaacgagagactgtatcaggcgcaccaacactacaacctctgacacacacactcctaacaaaacaaaataaatgctgttaagtggaccagcataccattctctctcactttagttctcgtggctcacacacacccacccacaagaaagcagaagaacaaaagaaacggaaaatatattatgtatagcctcttatgtaattcaagttatatattatgtattgtagaaagtattcggtgaaaactattaacacatatttgagtaagagtaaactgttgtttgactctcagaactattactctagttgaaattgattctacaaattatttttaggttaattaactttttaaattatttgaaattaaccaattaactcttcacagttagatacaataaaattcatctacttaatttgacgtcaaacactggcatgtgacccatacgtgatgtactcttgagggtaaagtcgtgccatcaattgatagcaaagtggataaatttcatcgaatttgacgatgaaaggttaattaactaatttcaaatagtttaaagagttaatttagcaaaaaagtagtttaaagggtcaaacggctatttacccgttgaaattataaacacaactattatattgaaggaagggaataaatcttagaaccccggtgtaaaagtgaatgagtaaatgttctcaaccaaccttgtttcaaaatattcatatttttgtttcttctcgttttgtttttatagaatcaaaagagaagaaaatcaaacatggtacaaccaacacaaaatcttaaccacttgacatacaagctctttttatcttttctttcttttaattatctttagcaaaacccttttaaactctgattaccctttagctaaacccttgttttagagcctgtctaatcgggtctttgagtcggctaatcaaatctcgaactgtttttggggatttaagctgagaacttgaggttgcaggaagattccaggcagacagtgcaccgtcaagtctaacatttcttttctccctccttttctctctccctctctctgtcttacacataaaacacattaatttggtggtggtttagggtttcagagagcaataattaggaactggaccagggtgtgcaggccgtgggagaggactaacgtgtgcctgtcggctcttctgcaaaaggagtactctctctctctctctctctctctgagtgtattcatcactcatattttgagtacaaaccaaaccacctgttcctgtttctcaaatggatttcaggggtttggatgggtggtaggaaacacaaaccactttctcacttccaaagagaaagagtcccccctctatctctctatactttctgatattaatcttagactaattagctttagcttagcgaggtttatattctatttaaccaaattccagaaccagcttcttaatacttgctttcgatgttggtgcaagttcaacaatctttctccactttttacagtctaatttgtaagatgtaaggttaatttaagctctaatttagtagggccccctcttttttcttttcctgtttgagttccaagatattgtttttttcattttttttttggaccttcttctctcatcactcaccttcttacctctttggttgaattttgtgagatgggttggtgtgttaagcacccaaaaatatcaaaatcatacaaaaaatataaaaaaataaagaatattaatattaagttagtgattatatttttattaagttttgctcttggattagctaggtactgtacagtgaattagggctgagaaaattacaaaaagatccatataattagggacaactataaattagaacatgaagataggtaatgtaaacgcacttctcatttatgttttgggaaaatttacagtaccctttaaaaaacggaggctcagatcacggccaagtatataatagctataagatatcagatttta |
| *MdZHD3* | tgcatatatatgtttgtaactgcttcctggctaggttgcttttgtccattcatcttgtgacacaacgagaaaagaaaaaatcaagaagaaaattggaatccatttgaaagtatgagaaatctcaaaatttacaaaatgacaattcgcctaaatattcctatttgtaatgtctcataactttaaatgctctctagaaattatccaaataagaatagaattaaacaattaataaaacactaaaaactattaaccaatgacatgtggcaagcaaatataagtcaagttagttacaaaggtagttaaggattgagtttgttaagaagggtaatcttgtaatggagtaaggttgttagagggtaatcttgtcattgtggattatcaatggtttctatataagtatatataaccatttagtccttcatattgtaatcatcaaaatatccattacaaataaatcaaagtgtaattcttcccttcatctctcttcatttctgcttgattagtttgttcctaaagcaatcttagattagttaataaaaacgtcctcaaaacttaaatcctaataaaacattgtaaacaatgaaatttggccgaaaagccgacatattacagtaaagcagcgagtttgatgtgcgttgtcattcttttcgaaacgatgcgtcatgagctcaaatcaaacactaaaacactaatatgcaaagttttattcttaatgtgcaccttccatcatctgaactatatataatcttactccaaattaaaagaacaaccatgttccaccaaccatccctcgcccataactccctgagttggacgaccttgactttgcattgcatgatcgatcaataatattctccaaaatgtatttaaatgtgtgttgtgttcatgaaccaagacaacacttctaggagtaactttcgagaaattcactccggtttatattcgcagtcttccgttacggttaacccttgtttttactaaatttacaaatttgtcatgccatgtgtaaaataatttggatctttaatcattatttgtttaagaaataaacatagtttgtttctaaaaaaagccacattttttataaaaaaaagccacatgcatgttcacccttaaccttaaacattctcgagcttttagggtaaattttgttaccctagctagctatatcagttaggcgatggagcgtgatataaagtgataattggttgcattggctgactagcttgtggacatattagcatggctaaatcttctctgtatcttgctgaaattttggacatatcaatcagctgccatacaactgcataggtaatgcttattgattttattgttgtgtccaaatgccaccgcgaaatctccccatacatatattatcgctcaggtcccttaattatgctcgttcttttctgttttctgtatttttttctttcaatttgatacatgttagaggatacaaaatttaaaaagtggtagtcatagttattgtgtattactaattgcaataatattatcacaccgatatgttaacagcgtgcgtaatagggtagctgttatcacaagtggtcgtcatagttattttctaatactaattaaatcaacatattcaatttctaaaaaactgattaacagacgacgtttaaatcaagatcctatttccttttcgtcttaaaccttggcacaaatctaagctacgagcccccttataacaatccaatgaaaaagcaaggtcaaatgaataattttttggaatggaagctgaactaccttttggttctgccagaatttttttttttttttttgaaattttatcacaaaaaattaatattgagctgtaatatgtaaaattctattcttcaacagttacggccaacatgggcagttgtgtaaataagccaaccaaatagtaccacattggaaaccactgtgttttatatatactgtaattgcaaatatctaacacagagagaggagagaggaaaagagaaagagagaagggg |
| *MdZHD4* | tctctctctctctctctctctctctctctctctctctaaaaccatatagatgttcaagcagtcatgccaaagatgctgttgcagctgtttgtttctatctctctttcttacaaactgtgagtactttcatctctctctctctctctctccataaagctgtgtttttcgcaaaaacagttgaaaattacgacatgggttttggtgggattagcaccctaatatttagtactaacttcgagacaagattatatatactaggaacaggatggatacacatttaaagatgtagctacatagctatatatatgccttctaattttgtcatatatttgaaacatatttctgcctttttgtgtttttctttttgtttattttagaacagcagagattaattaacagaaggaggactcggaaccccaagaaagtacacaaatactcgtttgaaatcaatgagacgatgcttttgtgacagcttttggtattaaattattgaagattgtgtgtttttgtgagagaatatcaaaaaatcc |
| *MdZHD5* | gaacagagagcaagagagttctgagaaatctggggattttggaaattcgaaggattggaattgctaaggtaatttgaccgagaggtttaagtggaggttatatagtaagcagttagttaacgttggaaattatgggttagtgcaaaggtttagttaacaacggctgtttaagtggtgcaagtcctaagtaaaggcatggggttcattttaatcatcattgacgcctcggattgcagactcaacaatgattaaatggggcattgtttgggttaatatttgaatattgggcttgaatgaaggaaagcccaaggatgccaaattaaatgaagatttgcccgaaacccagcatcgagcccaaagacaaactgaagccttcttagccaatacacaagccacgtgtctataactgagatgacaagtgatggagaccgacctattatcagccaaaaagcactttccagtggcactacaagtaaaaagctgatgactcactaccctccaagagttttcgggcaagatcaaagccctgaccttctggccaaatcgcctataaaagaaaaacagacaacagagataaggacactcaaccaatcaaacaaacaaacatacaaattctgctctcaagccatatttgcaaccaaaaagctgaaacccacccaaattcagtccttttagcaatagtttccttagaaaaagctatctttgtctagtataaaagctctgctacttccccagtgttgtactatcgattccctcatgtaaacttgtttaccatccatcccttttagcatacaatccctgtaaacttaaagagaagtggttgcaagaggttcaaccttgccagataaggtgaaatcttgcccgagtctctttgtttgtcctctaaattagtagatctattgcttaatgcactcttcagcatgtattcaagtgatttccacatattttctactttaagagtcccatttgcatctggatctggttgacttaatatatctgttttcattaagaataacttggaaccaaacaaatgacttaaagggctctggactccctccattcgaacatacaagatcatgactaaaagtctttcgtcacaaggcaagaaaaagaacttaatgcagacttaacccatctgcgacattcctttgataacaagaagtttgagtaacttggggcgcaaataatcaacttaaaacatacttgttctacatctactatactgagagagcagtggcacacctaagcatttagttatttttgattgcgagcctcaaggcctacacctaaagccccacaaaggcacatttcagaactaacttcaagctcttattgcggcacacaagacaagcaagagcccgactaccaaacatacgcatccaaagtgccaatcccttggggagctttgttgagggccagagaccttctgcagagaaatctttgcccaaacaggatgtctgattcttacatataataatcaaacgaaagtaactaaaagtaaatgaaggaggcaaacttaagttttgtcaagttgactaaagtaatgtgttttctctgcatctaactcaactcagttttcataatttaaataaatttaatatattcttccatcgtttgtaaacagaaaaaaaaatgtaaatgaaagggaaatgtttggaggaggactggggaattgatggccacattctccagcctcttatcttcttctccagaaaaaagtacccatgacaagataacaaaaaaagtaaaagaaaattaacagttatttgaaataaaaaaaaatttaataatattaattactattctgtaatacataacacttggatgtaatacaaaccaacatttagcagtagtcatttacatttgtctgtctccggttgaggaagctttattgttggtgatgatcgtcatctttattagttcattgggttcaccagcaaccatgtagatcttcacttctccttaaaaattcagagagagagagagggagagagagaga |
| *MdZHD6* | agaatattgtaggttaaatttttccatctccagctatgcagggctaaattttaacaccttagcttgggaagcacatgttgtgagcccgccaaggcgcttgggggcttgctatatgtactctacccttggctaaatgtagccttaattctttgtagcagactcattttggcccttggttggagacatattttgttcaaattcggactaaaagttacctatagcccttggctggaggcctaagtctcaatggaaaggaatttttacacacctattttctccttatgcattatatttgttattaactattagaggagattcaacggacgaaaataaacagggcgtgcaaaagcagatttgcacaaaacacccatgtatataaggccaacaccaccatgcaacttcatcttgcacttggagataatgtagaaagacaaattgcacaacactatgcatgacaaccacttggcttcgaaaaacttaagttgttaaaatatggaaccaaggacggagccaccttgtactcaccagggcggatgcgcccactcacattggtttgaaaataaaacagattacatgtaatttcaacttccacacattctcacattctaaattgaataaattaaaataaaactaatagagaaaaataaataaaagtgtacatagaaggataaaacatgtgtttggatatcatttcattttgcttaataatttttttgtttaatgggtccattcaaaaaaatctttagcttcatccctgtatggaacaacgatgctatccagctaactgtgatttaaatacgcacattattacatgtataatcggtgacacatatgatccatccatgaagtatatggtattatgttagagaaataattgaagtccagaacgagaaaagttgagaaggtttaattctcaatgggaagtaattttttgcacatctacttttttaccatttactctgtgttatagctaattaaaaaggttaattctcaatgggaagtaatttttgcacatctattttttttaccatatactttgtttgtttatagttactaataaaaataacataaataaaaatatgttggaaaaagatgaattgacacgaacaacgatgcgcatatcaagctaatacaaccaactttatattgcacttggagagaaatgcagacagatgaactacacgattcgcatgccaaccacttggctctgaaaaaattaagctatcaggatgtagaacaatgatctagtccagctaatagtgacttacatacgtacatataatcagtgacatgtatggaccatgcgttagtgacttaaatacgcatatataaatagcgacacatataaactacgcgtgaatgataagttattaagtcaaaatcaattaaaatcaataaatgggaagatggtaaaaggttagtctctatttgaagtgaaattttgcccacccctttgcatttttaaacactttaataatttttagtaattaaattgaataaattaataaaatttaagagaccaaaataaaagtatagaataaaaaataagagtgaaaattatttccggaactcaacttagtggcataagttgctaaggtatatttattgcacaacatctaacccctagtggggtaagcaacttaaccatggattagaactagggttaaaagaacaagtggttaagaaggacaaccacaaaacccagatttttattatcgacagtcatcactgctcatcatcaacatcatcatccctctctcggctagctaaaccgtgtctctctctctctctctctctcgccaaaaaaacggaaccatctttatcactcctccatacctcccctcactttctcaacatcctcagagcttccatggcagctcagagctgtaatctgcacagaaatctaatcctaacgacttaaagcagctcccttttaactttctctgtatttgcttttgatttttgttctcattttttggagacccctagatgtttgagcg |
| *MdZHD7* | atgatgggtgacccactgagaagttctcgtatgagttcccagaaacaaaatcgtgagggcgtggtcagggcccaaaacggacaatatcgtgttacggtggagtcgagcccaggatgtggtaggggctcgggccaggatgtgacaatttggtatcagaaccaatccctgaccggaagtgtgctgatgatgacgtcggggcccctaagggggtggacaacatcccacattgtccaggggagtggatcatgtaagccttatatgtatattctcatatctacctagcacgaggcattttggaaactcactggcttcgggttccatcgaaactctgaagttaagcgagttcgcacgagagcaatcccatgatgggtgacccactgggaagttctcgtgtgagttcccaaaaacaaaaccatgagggcgtagtcggggcccaaagcggataatatcgtgctacggcgaagtcgagcccaggatgcggtgggggcccaggccaagatgtgacaataaccgtctatgtatttgatacaatagattaaccaaacgactttagttttaattcattttttgctgagatgatctttacagagtgactcgtaatttgaacgattctaataataagtacaaagctctgtaattcaaatacgaagagttttaagtacaaaattatacttatctctgagtagttaaacattattcaactcaataattcatttcaaagtcctaataaactttcaaattttgatttacaccaagacaaaaggcgaagaaagtattcaaaaattggattagtttggtacaacacagaagaggagggaatccccactgccacagggccacagggccacaggcaaagcaaaacgatgcgcaccaggaggcactgggagctagctgtgtgctgaaaacccatgtactatttgaactgcgaatcttatgcgtgcaacctcactcgcatgacacgtacacgtttcgttgggtggcaggtgtccaaaatttgaatcgctgactcctacgaatatatattccaaattccaaacttttataagaacaaagaaaatttgcacaatggtatatgaattttttccaatttatcaaattatcacctagactaaagaaatttacagttgaccactcaaattttgaattgtattcgagcacatatgtgatatgtctgcttctattcattctcatttccgtcgaataatggctcgtgacaagaagatgtgcactttgtaaaagctattttcgtaatttcaatctaatatggtctaggtttgatgagttctgagttttttaggagaaaaaagttgtataaacacataatatatgtctattgagtgatgaacggtcccagtattttaagagttgtttggtatttaatttgaaacgtattttagttttttttttttttaatcaaatgaaacatttgttaaattagatgttgtataaaagagcagacgaagtttaaatccacaccattgtcctatactaacattcttctccatcactatagtaaagagtcacttccgttcacatgagtaattgtacataattgacaaagtcaaaaggaatatcatttatgcaattttctccaaaaataaaagaatagaatgaaaagaattatatgagagcctaatttattgctctcaacgaacacatgaaggcacggactgacatacgttacctcagattaaacccccatcccttcaagaagtgagagagattttttcccctagcttaattaggtggttgagaatattggtctgtgtttttatggagctaaaacaggtttaggtacaccactaattattggattaaattcatatcagatatctctgcccccactaaacaccccatctatatatttgtcttcacttctcattacatccttcatcaccccaatcatcctccaaaaccccatttaagttttcaactttgagctttcacaccctaagaacaaaaccctaatcagtttcttatgcttcttctcgcctaaagtt |
| *MdZHD8* | catctgtatatgtggtggatataaattgcagttagcatgaaaaaacacatacatatgtgattcctgacatatattcattgtgtgacagtatgagctaatggctttaggcttcatttgcttggctacatgtggactggagaattagcagatggctttctgaacaggttttcagtgtgtggttggaggaaattgtatcattgcattggtagtaaccaatactgttggagctagttagaattttatgcatgtaatccgattttttaattaagtagttaacagaaattgtacgtaaccctaattagatatattaattaattaaactgaagaagaaaacaaaatcgtagcaactgattgcaatgttgtcgtaagacaaggaagtgagatggattttcttacctattgatgtataagtacaaaacatgagaagtggcccaccttttgttccactttcccaacgtgaattattggatcaaacccataccacctctgcttcacgggtccccctcaactcatgatctgaagaattttgctagcttcttgtgtaaatttgtatttgctccctggcttggtcgctttaattaatgtctaaacatcctatttatagtttctcatatctctaaaagcttcaagagtccaaataagacactgaaaaggtcctttaaaacttaaaaacaccaataaaatattgtaaactatgaaatttggtttaaaaaaaccccatatatcacaacaaaatagcaagcttgatgcacgttaccattcttttcaaaacggcataccataagctcaaatcagacactaaagcttaaacctacaaagttttattcttactgctcaatttgcaccttccgccatatgatctatatcatcttactccaaattaaaaaaaaaaaaaaaaaaaaaaaaaaaaaaaaaaaaaaacaaccatgttccatgaaccatccctcgcccataacttcctgagttggacgaccttgactctgaattggatgatcgaacaataatgttccccaaatttttttattcaaatgtattgtttcatgaaccaagacaatacatctaagagcaacttttatttcaagaaattcatactcgtcactttatattgcaatgcgtataacattttatttattccatattttaagttcatattcacagtcttccattaacacttatgtttttactaagtttacaaatttgtcatgttattgtctcgtgaattcatttggatcttaatcattgtttctttaaaaaataaatagtttgtttctaaaacaatgtactctaatcaaaatatttaaaccctaattattttgccattgtttcacccttaaccttaatcattctcaagcttaatgttagttagggtaaatttgttaccctagctgtatcaattaggcgatagagcgtgatataaagtgacaattggttgcattgaacatttgaatgactagcttgtcgttatattagcatggctaaatcttctctgtatcttgccgatcatttcacaaagattctagacatatcaaacagctgcatgccatacaactgcataataaaatgcttatattgattttattgttgtgtccaaatgccaccggaaaatccccccatgcatataagattatcggtcaggaaccttattacatgctcgttttttctgtttttgtcatttttttttcatttttcaattggatataaatttatcataagaaataatatattactaatcacgttaatattgtgtcaacttaattacttatacgtgcataattacgcaatcgaaaataatattagtagtgaccctttcacctataaattaacgtatcatgaaattctattcttcaacagttacgaccaacatggcactagtgtaaataatccaaccaaacagtaccacattggaaacctgtgtctatttttacatactgtaattacaattatcaaacaaatagaagagggagggagggagagagagagagagagagagagagagaggg |
| *MdZHD9* | aagttatttgtttgtacaacatgcaaataaagatttatacgtgtgttaactatctttttcgatcaccgtaattgctttaagtaagtcagttgtttgtattataacaggagaatgaagatttattcacatttgagcaatcttttcaattactgtgtttgttttaattaaatcagatgtctgtattgaaacagacataggaggatttattggcgtttgctttgccttttttgatcactgtggtttgttgaggtaagttaaatgttcgtattataacatttgtatgaaacttaattcacattcgatatttgcaattgtttcatgtaagttaattgtctgcactataatatatgtagctaatgtctaatttatcctcacattgttacttgcacgttaattatgtcctggaaaaaatggtcaagactaagtctttatccttaaattcgaacgattagtaaactacaagctttaattgtttcaattcaagaaataatgatttaatttctttacaatttcaacaaatgtagcacgtgaagtgcacatccctggtgttttcatcatttgagttatttgtatgttttcaaacctattttttgtctcattgcttaaacgtgaagatctattcgtgtttgagctgctttatcgatcacagtaattgtttcagataaatcatttgtttgtattataatctattcaattgaaaagtgctaacatttaaacatcccaaaattccaatttaccctcgcattgtcaatctcacgaaaactacatagactacaaagtataactttgctcaaaccatgcattcaacaactcaaaagtctcaaatcatgatttgactaatgaattagtagtatcctaatccttgtggcctaccaatggtgttgaacctaattaagtaaaagttaattagtcttcttagtatgtgttttattaaggaaacaataagcaaaaagtgacgaatatcaaagccaagtttacgtcccttcaaggggtagctggaaatgggcaattttgggtcatttaagaggtgaagtgtgttgagataagaaatggaaaaaaaggggcaataatggaagaggggggaaggggttgtggtgtaagggttaggccccactgtggcagggcgtggggaggtctgaacatgacgttgtcggttgttttgtaatctgcaaacttgcaaagagagagagccagtgtgagcagtgtagtacactacgcacacctcctccctctcactctctctctctctctctctctctctctctctctctctctatttttacttgctatactagttgctttggttgtactcttatccttcccgctggcctcaccacaagcaaccaacctgtctcttgttggaagcacagaagcacaaaagcaccaacatctcaacggacccagctcaaacccagcatattcccagctctctctctctctctctctctctctaaaaccatagagatgttcaagcagtcatgctgaagatgctgttgcagctgtttgtttctatctctctttttcttacaaactgtgagcgtttcatctctctctctctctctctctctctctctctctctctctctccataaaagctgtgtttttggcaagaactgttgaaaattacgacatggccaggttttggtgggattagcaccctaatatttagtctaagttggagacgagaaatatacattctaggaacacgatggatatacatttagagtttcagctacatagctatatatatagcttctaattttgtcatatatttgaaacatttttcttcctttttgtgtttttgtttttgtttattttcgaacagcagagattaattaacagaaggacttggaaccccaagaaagtacacaaatactggtttgagatcaacgagacgatgatgcttttgtgacagcttttggtattaaataattgaagattgtgtgcttttgtgacagaatatcaaaaaatccatggattttagagctcaagataacgaa |
| *MdZHD10* | acaatcaggaagctgttttaacaactttggactgagcttcctcctaatattctttcagttttacgggataattgttgcttaattagttctttggctttttcttccatgtgactgaaaatgaaagtatggtaataccatgttagacaattattagcttaaaatttacgtcgtcgccgacatatggaccaatcaatatatatcaaacatttataggataatatacgtacagattacaatgcatgtgatagctaatgtgtttagaaactgttacgattttttaccttgttttagcgtcgataacttgtaaattatcgcatagacgagtaagcgaagagattcaatacgactaattaggacactctctcacttagaaatcatgtaaggctaggtgaaaagtggaatgactcgaccatcaaaatacaagagggtatcaatcttttgttagaacgacatggtcgattgagacaacctctgacaacactcactaacaaaacaaaataaatgctgttatgtggaccagcataccattgctctcactttagttcccttcacaccacccacaaaagaaaattaacaaaaaagaagaccaaaagaaaatctcccctctaattttctctttagaaaatttgttatgtattgtcattgaaatactggcgatcctcaactgttaatgtaaatatagaaatgacttcagttaaaactagcaacacacatataagtgataattacaaaactgtgacagtaaaaagaaatacttttgacataatacaaaaaacagagagtcttaatcactcaacatacaaactctttttatctttgcttttttcaataatctctagcaaaacccttttaaagtttcattaccattttagctaaaacccttgtttagagcctgtctaacggggttttacgtctgccaatcaaatctcaaatagtttttgaggatttaagctgagcacttcaggttgaaggaagattccaggaaaacagtgtaacgtcgagtctgacatttcttttctccctccttttatctctctctcttacacataaaacacattaatttggttgtggtttagggtttcagagagcaataattaggaactggaccagggttcaggctgtgggagaggactaacgtgagcatgtcggctcttctgcaaaagaagtactctctctctctctcactgagtgtattcatcacccattttgagtacaaaccaaaccacctgttcttgtttctcaaatagattttatggggttggatgggtggtaggaaacacaaaccactttctcgcttccaaagagaaagagtcctctctctctctctctctctctgagatattaatgaatcttacattaattagctttagcttagcgaggattatactctccttaatcaaagtccagaaccaggtaataatacctgctttcaataatggtggtgcaacttcaacaatctttctccaattttacagtgtaatttataagaggtcaggttaattttagctctaatccagtaggccccccctctagtttcttttcctgtttaagttccaagatttatcttttattttgtttttttgagctcttctgtacgtgagtttttcaatcttttaattcatctaatccatcaatatatctctctcacccaccttcttacctctttggaaacagggttgaaattttagtgagatgggttggtggatttagcacccaaaaatgtcaaaatcagaaatacaaataaaaaaataaagaagaataataatattaagttagtgattatatcttcgattaagttttgctcttaaattagctgggtactatagtatgaattagagttgagaaaattacaaaaaatccataaattatgagaaaatcataaatgagatcacgaagataggtaatgtaaacgcactactcatttatttttaggaaaattctacggatgctgagatcacggcaagtataataatagttacataagatatcagatttta |
| *MdZHD11* | ttgaagagtaaaaaggtactggactttgtcttcttgacaaagaaggaagtaatccatgtggaactagacttctttaggcttatggttagacgctggagcacagaagcccacaccttcgtctgcgcttaggcgaattcacccctaccttggaggatgtggccaacattatgcatttttctatcttggggaacgtagatccctttcactatacatgtggctgatgttgagatattcaaagtgctgcagaattgagcccctacttccttgagtaaggcgattcatttcaacgaatggattaaacatttttggcataaattctaggaatcaaactgcaagttcgaagctatgatatgtctgtaagaacatagacgcatgctgccttcgattcttgtgattgtgcggggtcacgtagttccgttaacctcattattccttggtttgttgtatcacgaggtaaatgagatccatgctttcgaggagcaggttgtcgacagcgttccaatcaaaagtttcttatgtgtaagcttcctacaaatatttctcttagattagatccatgctcaagctgtggtccatgctttcaatttcacagttcaacaagactaggtgaaagttatttttttaatttgatttcaaggaggtaatcgactgtatcaacactagatcgaagtcaccaccatgtgtgtattttccctacagttagcctaattcgactgaagagtctcctcgaacaccacaaatgggtgcgggctgcacgacaagtgaactatgcggccaacctggtctcactaactatgaagaagacacgcccgaaaaattgggtcaaaatacccccatcttctttgatcttcatgctgtctagtgatgggctgccttgctccacccccccccccttttgtcttcatagtcatcgtcgcctttccctttctattttctgtttcgttgtagtgtcttatatttgtttctgtcttttgggggccttggctgtttgtgtggcctgcctgccttttttgtgttttcgtggtttgacacttttgaccttatttttactgaaagtttgtttgactaaaaaaaaaaaagaagcttaccaattaggccttgtgtgtgtaaacttatgcattgggatgggaagggaaaaatatgagatagaaagttctaagaccaacattatttccctttagaaattggttggtaacaggtcttgtggaaccaaaggggtgctgttgcaccccacaacacaccaaaccaaagacgaagagtagtattctagtggtgttttttgttacaaaaacaccctcccaatattttgctctcttttctcagacagaaacacatgaaaaaattccaatacaaaatgcaaaattttgacgacaacaagcgcgtcacgcatgtcggaaacgagtgcacgtagcccagctgccttaaaagtcgacaatcacccaccctctctcccccgtacgcgacaccccacgcgcctccacgtctcttcctgctggaatgtgcccagcaccaccatccccgcttcagcgcgtgggacacttttgaacgaaggaattaaatatacaattaaataattctcttgtttttatttccagagtataaacgacgcgtaggggcagcggtggtctgtctgacactcccccacagctctctgaaacttaccgcgcggtgtaaaaacggagggtgaaaggtgagagtgaccggttcaaccgggagtagtcggttgggagtaggaaatgggaaagtatatcaagggctttcagggagccaagctattttgtttgcagggagaggtagtgaggggactgagtttatacaaaaatttacacctgacacacactctctctcactctctctctctctccctctctctaccttctctctctaaactcactcagccctctctttatcttcttctttctgggttgattctctttcattaattggtggggtttttccgtttttctctaattttaatcaatatatattaattaattgag |
| *MdZHD12* | gttctagtcaatcttttatgttttgttaattgatttatctctcttcttttgttaggtaatttatattttttttcttatctagttgtaatttgaaaataacgaataaatttatttattggtgagaaggtaataaccaaacagttactgagactatattagaagaatgtgcaaagtctcactaacttgttgttgctatttaataccacagtcaagtagtattcctccacttgagtgagaggctaaagttcgattttcactaaaggtaaatttgaaccacattattgctagttcattgtgaagcttagccctctctcctaacttagtgtagacaatatcctttgttctgaaaaaaataaaataaaataaacagctaaccttaatttttttttccttacacttgtaaactcaatgccttccaacaaagtggagagaactgtccttagggttgaatttgcattgaatcccttcaaatatcttatccctgtttccaaacatgaaccttgcaacatttgctacctcactctctggtctctgccatagagaatactggttaataagtgggtattccccactctctgccatttcctttttctactttcatgctaatagtgagaaagtgaatcctaatcccacttttaccacaaagataaaaaagaacaaaacaaaagactgtttccacataaaaggaaaacgcaaaaacccaaaacaaccaatcaaactccatgctcttaccaatacaattacccaaggaataaatgacaagaaaaaaaagcagaggctaccaaacaaatcccaaagaaaataacctgggaaaggtttaaagagttaaggccttccatagtaactgtaaaactgaatgtgatgtcaataatataaaaatcacaatcatacaatagatcaaaatatacgagtgggtgattcttatttttggttcaatatatcaaacacatgaatagttgatttatttgtaataaatgacacatatttgagaaataaaaagagaacaaccaatcataaattaaagaaaaagaaaaaaaatggtcccacgattattatatagaactcatatataccaaactcgcatcactctaacaataatgttatagaaattaaacttagactgtctgattcttatatataagaatcaaatcacttaaactattgtggaacgcctaaccactagttacgataaaatgaaagcaacttaaagtaaatgtaagacaaacttgagttttgtcaagttgattaaaacagtgtgcttcattttctccacttacttttaaactatcttttacgtaacttaaataaattaaatataattattatatcattttataaaaaacaaaaagaaaaagaaagtaaagtaaatgaaagggaaatgtttggagtaagactggggaattgatggccacactctccaccctcctcttatcttctcctctctaaaaaggaggtggattgtctgcccttccattttcatactctcctcatgcctttatgtttgtgtggtcacagttaagtcacgtcaacattttaaattcttattactttttgtttattatttctataaaaaaaattaatatgaaatattgacgtggcttaaccgtgaccatacatcacaagagggcatggaaagagtatgaaaataggagggcagacaatccacctccctctaaaaaagtacccattttacaagataacaaaaaaaatataaaaaaaattaacagttgtttgaaattaaaaaaaaaaaaaactaaataattatttactattatgtcatacataacacttggatgtaatacaaaccaacatttagcagtggtcatgtacttttgtctgtctccggttgaggaagcttttttgttgttggtgttgatcgtcatctttatcagttcattgggttcaccagcaaccatgtagatcttcacttctccttaagaattcagagagagtgagagagagtgagagagagagagagaggacatagagagagaga |
| *MdZHD13* | ataattatatatataggataatgctagctagatcaatttttagatcaaatttgcatggagtacgattatctcccctctttttttccctccccttccatccctcatatttgaacggtcatggttaagccacgtcaacatcttatattaattttttatagaaagagaaagataaaatagagaatgtgaaaggagggcatgaaaggggatggaaagaggaggggagagaatcctactccaatttgcatatcatgcgacgtgttaccgacataaacaacttgttaattaatacttaagtaataattcaatcatcaacaaccatatcatatagtttataaaatatggtttaaaaagagacattacatatatatatatatatatatatatatatatatattatattactgtcatgcatgtcattattttatctaataattggagctcaattaattaggcccttaggagtggagtttgagagtgtgaagattgaagacaaaggcagaagaagaaggtacacagaaaactttttgagttcaaataaagtacaaggcactcatattatgttctttaactaattcacttttaattttattcgattaaaagttaaaagaaaagtgtggagtgcaaaataaaaacaatattgcaaaaagaacttcccagcattgtgaaatatgtcagaggttttgctaattttccattgagacaacattacttttcataattcctatttggctctctctctctctctctgtgacaattggcattgtattgcttttgtcctttccactctctgacacacacacagcgctatatgtggcgtaaacttgcctgacagtgcaagcaaagctttaagcaagcaagcgcccccacttttttttttcttggattatgggtgctctctcaagtttcaactggtatggtttctccaaagctcacagtgtattcctctttgtctgtaaaaaagctatgcctttaactctctctctctctctctctctcgtctttcctaattgggcccctcatcaggactcatcactagtgagtatgtaatgtaagctgatattaatacttagtgcttcactagcaaaaccaccatcaaaactagtctctccacggttaagcttagctgtacctattctgtctctctctctctctgagtctgttctcctccaacaactgatctcttataatttcttgctttgtttctttaccacccattagctccatcagtttcccttcccttgctaacttaatcaatgatcagatttactttggtttgttcataaactactctatacttcaccattcatttgattaagctctctctatctctctctccctccaaattagccaaagaaagactgtttttctttttgcagcttctttgtgcctgtggaaaattggaaaatggaaggtgattgtttaagcagcgcatgagcatgaactagctatataccaacatatattctcgaagggaagttcatcctctaattgttttctttctttctttcattctttctgatcttctctactccttcagctatttctcgatcgattaatttcttttatgttgatcttccttcttgggagtcgttgatgagtgcgtggtgacctcctttactccctccctccctccctctctccttgtcccctctatctatctatctatctgtttcaaattaatgaaagaggaacactagtagtatttgctagcttggttctactactggtatacagctatttatggagatatatgtcaaagtggatgtggctcaagcgtgaatcaagacccttttaattaattgctatactgtgttctttaatcaaaaggattaattatatattggcaccatattcaaaccctccacttctgtttgtctctctctctcctctctctctcttgttccttctcttttggcattaaaataatagttagagaaaaaagaagtgaaagatccatctatctattctagctaaaggatagactgattgattaattaata |
| *MdZHD14* | cactgtgtccttcaaattatgtactctttgtactacgaatcctatgcgtgaaaactcatacgcatgacacgtatacgtttcgtattggcctgcaggtttccaaaattttaaatccctgacttcaacgaatatataagaacaaaaggaagttttgcacaatggtgtatgaattttttcatttttatgaaattgtcacctatactaaagaaatttccagttgaccactcaaattaaaaactatatttagcacatatgtctacttatattcatccacattttttttttcaaataatggctcgtgataagaagacatgccttgatccaagtttgatgaattaaattgagagagagtgttgagtttccttaagaacgagtccacccctaaggactttgtgccagcatccagcacatttatccactcaagtgaacaataatagactccggtgaacagtaataggtcaaagcatctccacccttaacaaaaaatagcctagtccattatattaaaatattaatttttttattataaaataataattttatttttaatttctgaatttataaaaaataaaataataataataataataatttctaaatttattaaaaagtttatttattttttaaaagtgaaattcttaattttttggaggaaaaaacttggaccgttgatctgtgatcggacggtccagtttaaaagtgaaattaaatttttttttactgttgaaaatccaacagtctagaataactagccgttggaaatccaacggttgcaagtgggccacgtcgctgagctcgggttgtggtctgagtgcgcctgcgtgcggaccccgccacctgatttcttgtctcctactcgcgcccacgcgagcgtgaaggccacgcgccagtgcaaaaatgcaaacaggccagtcccttggtcagtcaaaaatgggctgggctagagactggcatgggctgggcgccagtcaattgggtcggctggaggggatccactgggtcctggcctattttttcaactgggtgctgggcaaaaagtcctccggtggacttgctctaagagaaaaaagctgcaaaaacacatattttgacaacaataaggcgtctattaataaatgaacggtttcaaatgttttagtagttgtttgatattcaatttaaaataaacatataatttttaaaataatgaatataattaaattacttaatactcatttcttatataaacaggtaattatacaaaaaaaaataccaagcatgcctttagttgacttaacgaacaaaaatgctcttgttaaaggtgctcgatcgtgaagtttgggtgccaaattgtaacacaacataaaagtttgggtgataattatcaaagaaaataagttcacatgggcaattgcacacaactgaaaaggtcaaaataccatttatgcaattttgcctaactaaaaaaagatcaaatagaaagaattatatgagagcctaatttattgctctgaacaaacacatgaatccacagactgacatacgttacctcagattaaaccccatctcttaagaaattttagtgtgatagattatgtgtcgtaatacaagtgattgacttttttttttaaatatctagttatttatattataacatttgatatttaaattttattttcgatacattgaaaaatctcttcaagaaatgacacagaatttttcattcttaagtttttcccctaggttaatttggtggttgagaaatttgtctgtgtttttatggagctgagacaggtttaggtatgccactaattatcgaattaaatctacatcaaatatctttccccacaaacacccatctatatatttctctccacttttcattacatccttcatctcctcaatcatcctccaaaaccctattgaagttttcacctttgagatttcactccctaatatcaaaaccctaattaattcccaatccgcccgtcaatttcttatgcttcttctcgcctaaggat |
| *MdZHD15* | aaaggtgtacaaaaacagagttgcacaaaacaaccatgtatataaagccaacaccaccatgcaacttcatcttgcacttagagcgaaatgtagacagacaaactacacaacactgtgacttaaatacgcacttagagttaagttgtcgaaatatggaacaatgaagcaatccagctaactatgacttaaatacgcacattattacatgtagagaaatggtaaagggactctcttaaaagtgggattcttcatggactctctgccatctcatattttttgcacaatattttataatgttggcatgggaaccacgttaaactgtgaggtgacagagagtctatggagagtttcaattttgagatagcccccttagcatttctcttacatgtataatcagttgctcatacgatccatggatgaagtctatgctattaagtcaaagaattaattaaagtccataatagaaaaagtttagaagggctaattcttatgggaaataattttcgcactcctattttatttttatttattttttactatgcaccttgtttgtttatagctactaaaggagaattaacaaacaaaaataaatagagtgtgcaaaaagagaattgacacaacgatgtatatcaagccactaccaccaacggtccaactccatattgcacttagaaagaaatgcagacaaatgaactgcaaaactcggcatgacaaccacttggctctgaaaaaattaagctgtcaagatgtggagcaacgatgcagtccagctaacagtgacttaaatatgcatatataatcagcgacacgtatgaaccatgcgtgaaggataagctattaagtcagaatcagttaaaatcaataaatgcgaaaatgtgaaaaggttaatctctatttgaagtgatttttgtacatccctttgttttttttttacacacactattaattttagtcattagattgaataaattagtgaagaattaaagagaccaaagtaaaagcatagaaggaaaaattgggtttatggtaattatttcggactcaacttagtagcaactatagcataagctgctaaggtatatttatgggagcatttttgcttactactctaaacctttgtgtatgctcaccacacaaaatcatctgccacgtgtcatttcactttattatggttacttttttcaaaaattgaaaaagtaacatttaaatgaaagttaactagagttagttaaaaagacatatggcaaattattaggtgtatggtgatcatacactatagttaggttagtgagcaaaaacaatgctacttttaccttatatttataccataatttgtattatctctccaatagagatggaacccacatgcgttggtgggccctacatctattagagagattgtaaaaatacgtggtacgtgtaacattactcgagcaaaaatgcttcctatatttattacaaatgttactcttactacatatttgtaccatctctaatagagataagacttacatgtgttagtagacaatatctctattagagagatggtataaatgacggtacaaatatgtggtaagtgtagtatctcacaaatccctttctaattacccaatttatagcacacaacatctaaccccaagtggggtaagcaacttaaccatggattagaactagggttaaaaatacaagtggttaagaaggacaaccacaaaacccagatttttattatggacagtcatcactgctcatcatcatcatcaaccctctctcagctagctaaactctctctctctctatccccccaaaaaactcaccatctttctcactcctccatatctcccctctccttctcaatatcctcagagcttccatggcagctcagagctgtaatctgcacagaaatctaaccctaacgacttaaagcagctaccctttaactttgtctgtttttgcttttgattttcgttctctgattttggagacccctagatgtttgagag |
| *MdMIF1* | gtgacatgtagtgttccacgcacactgaaaaatatctcactaaaagctcgtttgaaagtgtttttaaaatgattgaaaacgcttttggtgaaattcattttgggttccaaaaaaacttcatatactttatagaagaagcactagatatgtgcttcttgcaggaagcagttcaagtattttttaggatccacttagatttttactaaggattggtttcaaaaacattttcaccaaaagcgttttcagtcattttaaaagcacttccaaacgaaccataatttcatatacattatttgataattaaatggtgatttcaatttttttgttcacttcaaatttcatttttcttacatatattagtacttaacttcaattcaaaagctaaaataagtgtgcaaaaagtgaagaaatgtgtgcataaaccatttcatattggcaaaatgtaaataaattttttatttttcttgcattagtagttaactttaaattaagaattgattttttttattcgtttaagggcagatttggggttgctgattttttttattcgtttaagggcagatttggggttgcctaatttttttgaaaagcaacttcttaatatagttggggctaaaattgtttggtaaacaaaaaattaagtgtttttttttatttttaaattaagcaacttcttaattaagtgattttttattttttatttttgataagaggcaggtaaccagtaccaattaatgaagctttcttattgacaatcctaccccatatctttcttctaaacctgaagcaccgttttttgctaaaaacacttttagcattataatttacaaagcacttatctacaacaaaagcaatttaaaagaaaaaaaaaagaacaacaattccaaactcgctctcaatatctgacaaagacggttgagatatcatttcaacaccaatctagctagctaaggttttcatttaggaaccagatattgtagctattagatcaaattttaatggtccaaatgcattgattagtggggtcagcatggttggttaacatataaggacaaaaaaattctactttgaattccactgtttattgaccactattgatacgagagagagacacgtttacagtacggaggaaacttttatatagccgtccaaaagaaatggttgcgatcttgcaaccaaacttctccaatcttctacttttattttattttattttggcatattgcattatgaaagttgcgatcaatttctttgtgacttgttacacagtcattagctgacaacgggaattcatgtcgattgataaaatctttgctctcttagtaaaacccaaaaggcactgaaaattctcatagctggtgtttttacgacaaatgacgtgttagtaagttaatttgtaaaatgttaaaagagagagatcggaatggagattgaactcacaccaaagtatataggcattattgatttcactactacgataaagcatcagctgcaacaaaggttgcttaataggttttgggcttggctcttatagatgaagagaggttttaagtttgactcttattgaataaacgatgttatctataatcaggagaatgagtgggtttagccttacaatgagttaacaataatgtggttcgattcgcctttggcgaaaatcaaacataagaactctcacttacgaataaaaataaatatctttagaccatagcactaatttattatgatggtctcatcatgtaaattatccaaaattcccaccacataaatacattttttatataaaaatattttaaaaaatgttcaagaccctaacccatataaatgtacctgccaggtttagggttaggcaccaaactaccaatacaacgccccacgcactcccctctatccttgtttggatgattaaagtactgaattcatagagtaccgaataccccttattgagctcacaatccaattacattaacacacaggcggattagggttttctgaagc |
| *MdMIF2* | tccgattatgaaaattttacagtgaagataagttataaatgagaaaatgagctctccccctaatgacaatatctatgtgatgaataaccgactcccaagttttgagaattaggtcactcaaaatcaaacacaacataaaatccaaaaccatccaaacgccttgaatgcatgatgtccattttttttttcctattatatatagctagctttgtgaatttcagtcacaacagacgtacgttccacatcacttctcagcagatatacaattaaattagtatatgaaggtttcagctgttaaatttcccctcacggacattgtcatcctagacctccttgtgtcgtcaaggacatgggagattccagatgttcggcagacttgatatttagttaaattgtggcaaaaacgcaaagatgtatgcatgcatgtggatcaaactatgagccacgtaatgaacaaaaatggcaaaaagttggcatagtcctccattgatatcagataccagcattacctaaccacatgtccgtcgctgttaacatgttacggagtttctttaataaaaaaattcgtctcacacttgtgcatggatgtgcaaatcagctctatagagcacatgaattcattttgcttagtgtaatttgtcgggtacaattggcacaaaataaaagaacatcacgtataaaaagcatatgaagtgatgtaggttatgtaatgtttgctcgtgttagtttataattacacgggcaaagagtatcatatcgatataaataggaatggaatcatcatttgtttttgtaattaatcaaaagccaacagtgtgcactttgtttatgtgtgtgtggtttgagattggatgaatccgtgacataattttgggaaaaccatcatcgatatattgcatggcaccaactattgtcttctctgtttctgtaaacattaaccatcaaacttttttttcttttgggtaaagatcaaacttctatttttgtttatttattctgcatatataaatcatgaaattgaattattaccttgtgcaatgcaagctaagtaacaagaagagaattataattgtagcatttgcaattttattttccgtatagggttggcaaggtgagatatcattttccatcgagttctggggtggtgaaactagactttttttggtcgttgaggaagctaagctagacaatttctattggcatcgtatcaatgaaccccacaaaacaatcgttttagtgccacttaagggtccacactgtacatgcataattatggtttatgaggtcttaatttctgcttagctggtattctatattaaacccatcgcagttatggttgatagaaaaagctttctcaacatatttagcatctaaggttttgccttatttacacacttcagtttccataattttattttatttttctttaattttatgctagtgagttgtgaggttgttggaccatcagttcacaacgaccatttttgggtttacaactaactagctagtaaacaatgagattaaaacattagtagttatgtagtaaaaaggtggtcatggatcgttgcccccccaattcctttcaatagaaagataaaaaaaaaattaccgactagcttttcttctaatgatatttagtatatatcagtaaaacaatttataattaaacaaaaacaggtcgattactgtgcttaccaatagttaagactactaaagcaagaagagagagagaatattacatgctaatatgctataaatatatatctagggttagggcttggtcattaccaccacataaccataagctcttgtttcctttctttctctctctctttatcactctctaactcacctccaccaacttctaatttgccatcttttgattctgagtcaaaaaaccagtgaggaaatttcagcatagtttcaagggggtttggggaaatcaagagacgtaataaaatcgagatctgaaacagggacgaattaatcagaaacttaa |
| *MdMIF3* | atgttgaaatgttataaagttctgattgtccacgatgtgatgcaaagagaaaagaattgtggtcattttcaagagcgtgggcaatgctcttaataattaacgaccatcctttccgaaatttctttatgaggattctgagaattttcaaattatgttcactcatcatacatcgtgcaatcaattttcatcatatactattcatatttaatttaaaataaaaatatttaaaataaattttaaccgtacaatgtacgatgaatagacacaatttaagaattctcaagatgctcgcaaaaagagtcaagagaggaacctcattccttaataattagatcttaggtacttagacaagagttgttgtcacagttgcaagggccctggcaacctgtctttcacctatcaatttggacccatgaaaaccaaaaaaagaactagaatctggataattgattggtcattctgtgctaaacaatcctcactaattcgtgtacattatctaatagccacattattgtaacatatcttataattaaatgttgacaagggaaatgattatattttttttaatcttctgcaatttttttcttaatttcaactgctaaacgtgtttgatttgttcacttcagttcaaactttatttttctcaccttcattcttgactttaattaaaaaaactaatgtgcataaataattttctacctataaaatgtaaatgaaaatttcatttttctcgcacattagtagtaattaacttcaaattaagaattgattttttttgttcatttaaatgtctggcaagacagttgagatatcatttcaaacaccaacctagctagctagggttttcactttacattgtttatatgtaaggacaaaactttctactttgaattccactattgatatgagagtgagccacgtttccagtctggaggaaacttttatttaaacctccaaaagagatggtcgcaatcttttaatcaaacttcaccaaacttctacctttttattttggcattataattaaagttgcgatcaatttttttgtgacttgttacacagttcctaaccgacaacatgaattctatgtcgagggatgaatccttgctctctttatcaaacccaataggcacatagaaaattccctcgggtaaaaacaagggctacttgatgcagtttattagtattttatttatttataaataatagattttaaattcaacttttatggataactaaaagttttaaattcgattcttataaatagcaaaggttttttagttaaaattgtgcatgagatttgcataatacattaatttggtccctgaaatttaaaatcaatagaagttgtccttgagattgtccactatctattattttgatcattccgttaaaaaactccgttaagttgagggtataacacataactttggtccctgagatttgcataacacataattttggtctctgagatttgcataacacataactttagtccctgagattgtccaacattcatcatttttggtcatttcattaaaaagctcagggaccacttaacggaattttttaatggaatgaccaaaataatggatggtgaacaattttagtgaccacttctatttattttaaatcttaaagaccaaagttatgtgttatgaaaatctcatggaccattttcactaagaagccaataacaaattcgatatcaattaatttaaccaaatccctctccctccttatatttctttcttctacgctagtggctagctagtggtgtaccacgcactaacctatttaaatatacctgctagggttagggttaagtcaccaagcgactaatacaacgcgcccccaacccccctcgttttgtgagctcctcagtaatccttgtttggatgattaaagtactgaatacatagagagtgcatagtaccgtacttgttgctctcaaacatccaattacatcaacacaaaggcggattagggttttctgaagc |
| *MdMIF4* | attttttccactacagctagcattgtgaattcagtcacaacatacatatgtacgtaccacatcacttatagcagcacatatataaactaatatatgaaggtttcaacggttaaatttcccctcacggacattgtcatccttggcctccttatgtcgtcaaggacatgggagattgagatgttcggcagacttaatatttagttgaattttggcaaaaacgcaaagctgcatgcatgcatgtggatcaaactataagccacgtaatgaccaaaaatggcaaacagttggcgtagtcctccattgatatcagataccagcatattacctaaccacgtgtccgctaacacattttcatgttagggagtttcttttctaactttttaaccaaaaaatccgtctgacacttgtgcattcatgtgcaaattagctctatagagcacatgaattcattttgtttaggtgtaaaattgtcgggtacaattggcacaaaataaaataaaatcacgtataaaaagcatatgaagtctgtaggttatgtaatgtttgctcgtgttagtttataattacacagacaaaattatatcatatcggtttaaatatggttggaatcatcatctgtttttgtaattaatgaaaggcaaaattgtgtactttgtatatatgtgcgcttgatattggatgaatctgtgacataatttctaggaaacataatgctagggagatcaaaattttaaattaaatttgcaaatcagatgatgtgttaccaatagaaaataattatgttaataatgttagtgagaccaaaattttaaatcaaatttgcaaaccaggtgatttgttactaataggaaataagcatgcttaatcaacacttaagtaataatccaatcatgaacaactacatttgattttcaaaatttagttcaaatatttaatctcccaagcattatccttttgcaaaatcattattgatatattgcatggcaccaactattgccttctctgtttctataaacttaaccatcaaacttttatttttatttattaattatgcataaaccatgaaattgaattattaccttatgccatgcaagcaagataacaagaagagaattagaattgtagcatttgcatttccgtacagggttggcaatatatcattttccatcgagttttagggtgatgaaattaaactattttttggtcgttgatgaagctaagctagacaatttcctttggcatcgtatcattgcaccccacaaaaccatccctttagtagcactttagggtccacacagtacatgcatattatgttttatgaggtcttaatatttctgcttagctggtcattctatattaaaccaatcggagttacgattgatatagaaaaagctttctcaacatatttagcatctaggctttttccttatttacacagatcagatttcataatgagttttgaggttgttggaccatcagttcaccaagaacattttgggtttaccagtagctagcaagtaaacaatgagataaaacattaatagttacatagtaaaaagttggtcatggatcatgcccccacttcctgtcaatagaaaaataaaacaatataattaccgactaatttttcgtctagtgatatctggtatgagataaaacaatttgtaattaaacaaaaacagctggattactgtgcctaccaatagttaagactactaaagcaagaagagagagagaatattacgtgctaatatgctataaacatatatctagggttagggcttggtcataccaccacaaaaccataagtacttgtttcccctctctttccctctctcaatcaccaactcaccgccccgaacttctaatttgccatctttgattctgagtcaaaaaatcagtcagtaaattcagcatagtgtcaagggggttcggcgaaatcaagaaacgcaattaataaacaagatcttaaacaggtgtctaatttatcagaaacttaa |
